# Lyme borreliosis in pregnancy and associations with parent and offspring health outcomes: An international cross-sectional survey

**DOI:** 10.3389/fmed.2022.1022766

**Published:** 2022-11-03

**Authors:** Katherine Leavey, Rachel K. MacKenzie, Sue Faber, Vett K. Lloyd, Charlotte Mao, Melanie K. B. Wills, Isabelle Boucoiran, Elizabeth C. Cates, Abeer Omar, Olivia Marquez, Elizabeth K. Darling

**Affiliations:** ^1^McMaster Midwifery Research Centre, McMaster University, Hamilton, ON, Canada; ^2^LymeHope, Ontario, ON, Canada; ^3^Department of Biology, Mount Allison University, Sackville, NB, Canada; ^4^Dean Center for Tick Borne Illness, Spaulding Rehabilitation Hospital, Boston, MA, United States; ^5^Invisible International, Cambridge, MA, United States; ^6^G. Magnotta Lyme Disease Research Lab, Molecular and Cellular Biology, University of Guelph, Guelph, ON, Canada; ^7^Centre Hospitalier Universitaire (CHU) Sainte-Justine, Montréal, QC, Canada; ^8^Department of Obstetrics and Gynecology, Université de Montréal, Montréal, QC, Canada; ^9^Trent/Fleming School of Nursing, Trent University, Peterborough, ON, Canada

**Keywords:** pregnancy, Lyme disease, Lyme borreliosis, survey, symptoms, transmission, birth outcomes, child health

## Abstract

**Background:**

Lyme disease (LD) is a complex tick-borne pathology caused by *Borrelia burgdorferi* sensu lato bacteria. Currently, there are limited data regarding the health outcomes of people infected during pregnancy, the potential for perinatal transmission to their fetus, and the long-term effects on these children. Therefore, the primary objective of this survey study was to investigate the impact of LD in pregnancy on both the parent and their offspring.

**Methods:**

A seven-section survey was developed and administered in REDCap. Although recruitment was primarily through LD-focused organizations, participation was open to anyone over the age of 18 who had been pregnant. Participant health/symptoms were compared across those with “Diagnosed LD,” “Suspected LD,” or “No LD” at any time in their lives. The timing of LD events in the participants’ histories (tick bite, diagnosis, treatment start, etc.) were then utilized to classify the participants’ pregnancies into one of five groups: “Probable Treated LD,” “Probable Untreated LD,” “Possible Untreated LD,” “No Evidence of LD,” and “Unclear.”

**Results:**

A total of 691 eligible people participated in the survey, of whom 65% had Diagnosed LD, 6% had Suspected LD, and 29% had No LD ever. Both the Diagnosed LD and Suspected LD groups indicated a high symptom burden (*p* < 0.01). Unfortunately, direct testing of fetal/newborn tissues for *Borrelia burgdorferi* only occurred following 3% of pregnancies at risk of transmission; positive/equivocal results were obtained in 14% of these cases. Pregnancies with No Evidence of LD experienced the fewest complications (*p* < 0.01) and were most likely to result in a live birth (*p* = 0.01) and limited short- and long-term offspring pathologies (*p* < 0.01). Within the LD-affected pregnancy groups, obtaining treatment did not decrease complications for the parent themselves but did ameliorate neonatal health status, with reduced rates of rashes, hypotonia, and respiratory distress (all *p* < 0.01). The impact of parent LD treatment on longer-term child outcomes was less clear.

**Conclusion:**

Overall, this pioneering survey represents significant progress toward understanding the effects of LD on pregnancy and child health. A large prospective study of pregnant people with LD, combining consistent diagnostic testing, exhaustive assessment of fetal/newborn samples, and long-term offspring follow-up, is warranted.

## Introduction

Lyme disease (LD) is the most common tick-borne disease in North America and Europe, with known cases rising rapidly in recent years (1). Primarily transmitted through bites from infected *Ixodes* ticks, LD is caused by spirochetal bacteria within the *Borrelia burgdorferi* sensu lato species complex, also known as the Lyme borreliosis group (2). The disease generally consists of three stages (“early localized,” “early disseminated,” and “late disseminated”), although symptoms often vary and can overlap between stages. The first stage is expected to appear within days or weeks of infection, sometimes with a localized, characteristic erythema migrans (EM) (“bull’s-eye”) rash, as well as flu-like symptoms (3, 4). If left untreated, initial signs of Lyme disease progression can include multiple EM lesions, cranial nerve palsies, meningitis, and carditis, followed by inflammatory symptoms of late-stage dissemination, such as multi-system dysfunction, arthritis, and late neurologic and cutaneous pathologies (3–5). Even among people who do receive standard antibiotic therapy, 10–36% report persistent symptoms (6–9). However, despite the importance of timely and adequate treatment, rapid and accurate diagnosis is often lacking as symptoms can be difficult to identify (10–13), diagnostic criteria and physician awareness can vary significantly between regions (14–17), and current laboratory tests for Lyme disease are often insufficiently sensitive, especially in early stages (18, 19). As such, true case counts are likely highly underestimated, with many LD patients going undiagnosed and untreated (1, 20, 21).

A diagnosis or suspicion of Lyme disease is of particular concern for people who are pregnant or planning to become pregnant. Available research focused on the transmission of *Borrelia burgdorferi* from an infected person to their child *in utero*, as well as the impact of LD on pregnancy complications and the long-term health of offspring, has been both limited and discordant. Evidence to support the potential for perinatal transmission of the bacteria is drawn from multiple case studies of gestational Lyme disease where spirochetes were identified in the placenta and/or fetal tissue, including the fetal brain, heart, liver, lung, spleen, and kidney (22–28). In some cases, fetal transmission occurred even though the parent was asymptomatic and/or tested negative for Lyme disease by standard serological assays (22, 24, 26, 29). Additionally, LD-affected pregnancies have been associated with spontaneous abortion, stillbirth, premature delivery, intrauterine growth restriction, and neonatal death (30, 31), as well as a variety of neonatal conditions following a live birth, such as hyperbilirubinemia, hypotonia, cardiovascular and urinary tract defects, orthopedic and neurological abnormalities, respiratory distress, and rash (23, 24, 27–29, 32). Fortunately, there is also evidence to suggest that treating the parent for Lyme disease prior to or during pregnancy may reduce the frequency of these adverse outcomes (30, 33). However, in parallel, several cases of parental gestational LD with few pregnancy complications and no indication of *Borrelia burgdorferi* transmission have also been reported (34–37). Moreover, the frequency of fetal positivity is unclear and often not tested, a consistent syndrome associated with congenital infection has not been identified (38–40), and child health outcomes are rarely followed past the newborn stage (32, 37, 41, 42). As such, it is essential that additional research on the perinatal transmission of Lyme disease is conducted.

Here, we present a significant first step toward this goal with a large international cross-sectional survey of people who have experienced Lyme disease during pregnancy. The objective of our survey was to investigate the health outcomes of people with LD in pregnancy and their offspring, and to compare these to people without LD in pregnancy.

## Materials and methods

### Study population

Participants were eligible to complete the survey if they were at least 18 years old, had been pregnant at least once (regardless of the outcome of the pregnancy), and provided written informed consent for the collection of their and their child/children’s health information. Participants could reside in any country and could have been diagnosed with acute or chronic/late stage Lyme disease, suspected they have or had Lyme disease, or never been diagnosed with or suspected to have Lyme disease. We recruited participants directly through Lyme disease-focused organizations’ websites and social media platforms, as well as through the website and social media platforms of the McMaster Midwifery Research Centre.

### Survey development

Members of the research team, including Lyme disease research experts, a pediatric infectious disease specialist, and a patient advocate, informed the creation and refinement of the survey. Questions were based on available survey and questionnaire tools that have been previously utilized in Lyme disease research (43, 44), including the General Symptom Questionnaire-30 for measuring symptom burden (45). The survey contained multi-option questions, rating scale questions, and open text fields, split across seven sections: diagnosis and treatment of Lyme disease, suspected Lyme disease and tick bite history, symptoms of Lyme disease, pregnancy information, child health information, demographics, and patient priorities (Supplementary Table 1). The survey was administered online in REDCap (46, 47) in both English (available between September 25th, 2020 and November 22nd, 2021) and French (available between January 25th, 2021 and November 22nd, 2021). Before publication, the survey was assessed for functionality and content validity by members of the research team and piloted by seven people (affiliated with a Lyme disease organization or directly/indirectly impacted by Lyme disease) who were not involved in its development.

### Data collection and ethics

Survey data were collected anonymously, and participants were assigned a unique passcode. All responses were stored in REDCap and accessible only by the research members at McMaster University *via* a username and password. Ethics approval for this study was granted by the Hamilton Integrated Research Ethics Board (HiREB) (project #11222).

### Data analysis

Data cleaning and statistical analysis were performed in R 4.1.1, using Kruskal-Wallis rank-sum tests, Wilcoxon-Mann-Whitney tests, Fisher’s exact tests, and Kendall rank correlations, as appropriate. If a participant commenced the survey multiple times (as determined by identical provided dates and data), the most complete set of responses was included. Participants were not required to complete the entire survey for their provided data to be utilized. Information from all unique, eligible participants contributed to the analysis of parent health and disease symptoms; however, pregnancies without a valid date of birth (DOB), due date, or miscarriage date were excluded from further analyses as the timing of gestation in relation to the timing of important Lyme disease-associated dates was critical for pregnancy classification. Child DOBs were considered invalid if they were biologically impossible (e.g., child bitten by a tick in 2003 but born in 2020).

We classified pregnancies into five groups: “Probable Treated LD,” “Probable Untreated LD,” “Possible Untreated LD,” “No Evidence of LD,” and “Unclear.” Pregnancies were classified as “Probable LD” if the birthing parent was diagnosed with Lyme disease and/or had an EM rash before or during the pregnancy, with the LD either “Treated” or “Untreated” before or during the pregnancy. A specific date for post-treatment symptom resolution (if any) was not queried in the survey and, therefore, a comparison of outcomes from “Resolved” vs. “Unresolved” LD pregnancies was not feasible. “Possible LD” pregnancies were those where the parent had been bitten by a tick and/or experienced an onset of non-EM Lyme disease symptoms (e.g., recurring headaches, joint swelling or pain, facial drooping, muscle spasms or twitching, numbness and tingling, etc.) before or during the pregnancy (all “Untreated”). Pregnancies were classified as “No Evidence of LD” if the parent had no diagnosis or suspicion of LD in their lifetime and either no tick bites at any point or no tick bite prior to or during the pregnancy. No Evidence of LD pregnancies also included those involving parents who eventually developed Lyme disease but provided dates for at least two of LD diagnosis, earliest tick bite, EM onset, and non-EM symptom onset, and these were all after the pregnancy. When only a post-pregnancy diagnosis date was available, or multiple dates were provided, all after the pregnancy, but the child was noted as diagnosed with congenital Lyme disease, the pregnancy was instead classified as “Unclear.” Pregnancies with one or more critical date(s) for classification (including treatment start date) in the same year as the birth and with a missing month were also classified as Unclear. Unclear pregnancies were not included in statistical analyses.

If a date that did not impact a pregnancy’s classification (e.g., parent DOB) had an available year but was missing the month, June (06) was used as a replacement. Child health outcomes were assessed using all live births with a valid DOB, as well as using similarly aged subsets of children to control for the effect of child age at the time of the survey. We selected the subsets to be as large as possible and as balanced as possible (i.e., similar proportions of included children from each group) while maintaining a non-significant *p*-value (*p* ≥ 0.05 by Fisher’s exact test) across the comparison groups for child age.

## Results

### Participant demographics

**FIGURE 1:**
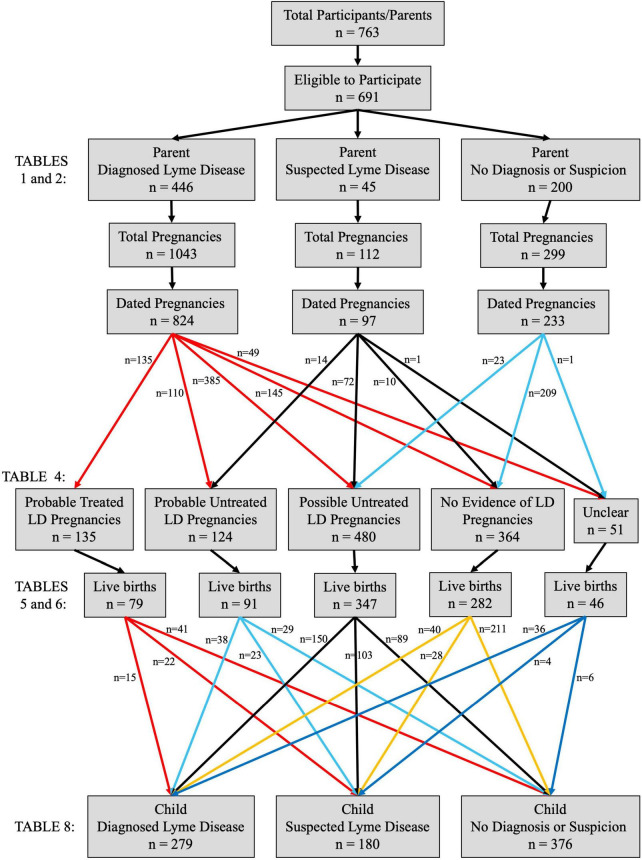
Participant groups and pregnancy classifications. Of the 763 individuals who commenced the survey, 691 were eligible to participate (at least 18 years old, had been pregnant at least once, and provided written informed consent). Most participants (*n* = 446) had been diagnosed with Lyme disease (LD) at some point in their lives, while some suspected they had or have Lyme disease but were never diagnosed (*n* = 45). The remaining participants (*n* = 200) had never been diagnosed with or suspected they had LD. Overall, the 691 participants had been pregnant a total of 1,454 times, and a valid date of birth/miscarriage date/due date was provided for 1,154 of these pregnancies (79%), allowing for the classification of the pregnancy based on the Lyme disease status of the participant/parent. Pregnancies were classified as “Probable LD” if the parent was diagnosed with Lyme disease and/or observed an erythema migrans (EM) rash prior to or during the pregnancy (may be “Treated” or “Untreated” prior to or during the pregnancy). “Possible LD” pregnancies were those where the parent experienced a tick bite and/or onset of non-EM Lyme disease symptoms prior to or during the pregnancy (all “Untreated”). If at least two dates were provided for diagnosis, tick bite, EM onset, and other symptom onset, and they were all after the pregnancy, it was classified as a “No Evidence of LD” pregnancy, along with any pregnancy in a healthy control with no noted tick bite at any point or no tick bite prior to or during the pregnancy. Fewer than 5% of pregnancies (*n* = 51) could not be classified due to missing or conflicting dates (“Unclear” pregnancies). The majority (73%, *n* = 845) of dated pregnancies resulted in a live birth and the Lyme disease status of the child themselves at the time of parent participation in the survey was noted for 99% (*n* = 835) of live births.

A total of 763 individuals commenced the survey (754 in English and nine in French) and 691 (91%) were eligible to participate (Figure 1). The majority of participants (*n* = 446, 65%) reported that they had been diagnosed with Lyme disease at some point in their lives (“Diagnosed LD” group), while some suspected they had or have Lyme disease but were never diagnosed (*n* = 45; “Suspected LD” group). The remaining participants (*n* = 200) had never been diagnosed with or suspected they had Lyme disease (“No LD” group). There were no significant differences across the three groups regarding their age at the time of participation (*p* = 0.16), racialized status (*p* = 0.33), pregnancy status at the time of participation (*p* = 0.87), total lifetime number of pregnancies (*p* = 0.52), and overall prior pregnancy outcomes (*p* = 0.24), although the Suspected LD group did demonstrate a somewhat increased frequency of miscarriages (30% of previous pregnancies vs. 22% in the Diagnosed LD group and 20% in the No LD group, *p* = 0.09) (Table 1). The No LD participants reported a higher achieved level of education (*p* = 0.09), as well as a significantly higher overall household income (*p* < 0.01), compared to both the Diagnosed LD and Suspected LD groups (Table 1). A discrepancy in household income was further observed between the two Lyme disease-affected groups (*p* = 0.04), with Suspected LD participants reporting the lowest overall salaries. Participant location was also unbalanced, with most No LD participants (82%) residing in Canada and more Diagnosed LD participants (56%) living in the United States (*p* < 0.01) (Table 1). Overall, 87% of participants resided in North America.

**TABLE 1:** Participant/birthing parent demographics.

	Parent diagnosed LD *n* = 446	Parent suspected LD *n* = 45	Parent no diagnosis or suspicion *n* = 200	*P*-value^a^
Age at participation, years [mean (SD)]	44 (10)	44 (10)	42 (11)	0.16
Total number of pregnancies [mean (SD)]	2.8 (1.5)	3.1 (1.8)	2.8 (1.5)	0.52
Currently pregnant at participation	6 (23/380)	8 (3/40)	6 (7/115)	0.87
Prior pregnancy outcomes^b^				0.24
Livebirth	73 (647/888)	66 (69/105)	73 (187/255)	
Miscarriage	22 (199/888)	30 (32/105)	20 (50/255)	
Stillbirth	0.3 (3/888)	0 (0/105)	0.8 (2/255)	
Elective abortion	4 (39/888)	4 (4/105)	6 (16/255)	
Identify as racialized	11 (27/241)	8 (2/24)	6 (5/89)	0.33
Education				0.09
High school incomplete	1 (3/310)	3 (1/30)	2 (2/100)	
High school diploma or equivalent	7 (21/310)	10 (3/30)	2 (2/100)	
Some college	13 (41/310)	17 (5/30)	6 (6/100)	
College diploma/certificate	20 (62/310)	20 (6/30)	24 (24/100)	
Some university	3 (9/310)	7 (2/30)	3 (3/100)	
Bachelor’s degree	34 (104/310)	27 (8/30)	30 (30/100)	
Graduate degree	23 (70/310)	17 (5/30)	33 (33/100)	
Household income				< 0.01
Less than $20,000	8 (21/265)	19 (5/26)	1 (1/91)	
$20,000–$34,999	11 (30/265)	4 (1/26)	2 (2/91)	
$35,000–$49,999	9 (23/265)	12 (3/26)	3 (3/91)	
$50,000–$74,999	18 (48/265)	19 (5/26)	14 (13/91)	
$75,000–$99,999	13 (35/265)	27 (7/26)	22 (20/91)	
Over $100,000	41 (108/265)	19 (5/26)	57 (52/91)	
Country of residence				< 0.01
Canada	28 (89/319)	47 (15/32)	82 (82/100)	
United States	56 (179/319)	38 (12/32)	17 (17/100)	
Other	16 (51/319)	16 (5/32)	1 (1/100)	
Heard about the study on social media (Facebook, Instagram, and/or Twitter)	76 (244/319)	59 (19/32)	68 (68/100)	0.04
Heard about the study from an organization’s website or an emailed newsletter	16 (52/319)	25 (8/32)	6 (6/100)	< 0.01
Heard about the study from a friend and/or family member	12 (38/319)	12 (4/32)	37 (37/100)	< 0.01
Heard about the study from a healthcare provider	3 (9/319)	0 (0/32)	10 (10/100)	< 0.01
Completed entire survey	60 (268/446)	60 (27/45)	50 (100/200)	0.05

Includes all individuals who were eligible to participate. Values are percentages (n/N) unless stated otherwise. ^a^*P*-values calculated by Kruskal-Wallis rank sum tests (continuous data) and Fisher’s exact tests (discrete data). ^b^Pregnancy outcome data by individual pregnancy, not participant.

### Participant Lyme disease symptoms, testing, and treatment

Both the Diagnosed LD and Suspected LD participants were more likely to have been bitten by a tick (*p* < 0.01) and experienced an EM rash (*p* < 0.01) in their lifetimes, compared to the No LD participants (Table 2). However, only 45% of Diagnosed LD and 61% of Suspected LD participants recalled a tick bite, and < 40% noticed an EM rash. Both LD groups also scored much higher on the General Symptom Questionnaire-30 (*p* < 0.01) and were more likely to identify as disabled (*p* < 0.01) and note that their symptoms impaired their work, social, or family functioning (*p* < 0.01) than the No LD group (Table 2). When the Diagnosed LD and Suspected LD participants were asked about their specific non-EM Lyme disease symptoms, they reported no significant differences, with similar rates of fever, pain, and cognitive dysfunction (Table 2).

**TABLE 2:** Participant/birthing parent Lyme disease symptoms, testing, and treatment.

	Parent diagnosed LD *n* = 446	Parent suspected LD *n* = 45	Parent no diagnosis or suspicion *n* = 200	*P*-value^a^
Resides in a Lyme disease endemic area	56 (139/248)	83 (20/24)	88 (43/49)	< 0.01
Any tick bite	45 (174/385)	61 (23/38)	21 (26/124)	< 0.01
Removed and tested tick positive for *Borrelia burgdorferi*	27 (3/11)	100 (1/1)	0 (0/4)	0.15
Erythema migrans (EM) rash	38 (125/329)	39 (7/18)	0 (0/19)	< 0.01
Any non-EM Lyme disease symptoms	99 (403/408)	100 (44/44)	NA	1
Fever	50 (203/408)	57 (25/44)	NA	0.43
Recurring headaches	73 (298/408)	75 (33/44)	NA	0.86
Recurring neck pain	72 (294/408)	75 (33/44)	NA	0.86
Swelling of joints	48 (195/408)	59 (26/44)	NA	0.20
Joint pain	80 (328/408)	89 (39/44)	NA	0.23
Muscle pain	77 (316/408)	89 (39/44)	NA	0.12
Fatigue	92 (375/408)	100 (44/44)	NA	0.06
Facial drooping	19 (78/408)	30 (13/44)	NA	0.11
Numbness and tingling	74 (301/408)	84 (37/44)	NA	0.15
Muscle spasms or twitches	67 (274/408)	75 (33/44)	NA	0.31
Brain dysfunction (including memory issues)	82 (333/408)	89 (39/44)	NA	0.30
Lack of joint/muscle stability or function	54 (221/408)	66 (29/44)	NA	0.15
Other Lyme disease symptoms	59 (240/408)	52 (23/44)	NA	0.42
Ten or more of the above symptoms	45 (183/408)	52 (23/44)	NA	0.43
Positive ELISA or Western blot	35 (128/368)	9 (2/23)	NA	0.01
Positive ELISA and Western blot	39 (145/368)	9 (2/23)	NA	< 0.01
Positive other blood test (e.g., PCR-DNA, EliSpot)	32 (119/368)	35 (8/23)	NA	0.82
Positive non-blood Lyme disease test	23 (104/446)	NA	NA	NA
Lyme disease diagnosed at stage I (early localized)	6 (25/392)	NA	NA	NA
Lyme disease diagnosed at stage II (early disseminated)	2 (9/392)	NA	NA	NA
Lyme disease diagnosed at stage III (late disseminated)	91 (358/392)	NA	NA	NA
Treated for Lyme disease	94 (383/406)	NA	NA	NA
Oral antibiotics	84 (322/383)	NA	NA	NA
Intravenous antibiotics	26 (100/383)	NA	NA	NA
Other treatment	54 (207/383)	NA	NA	NA
Tetracyclines	82 (271/332)	NA	NA	NA
Cephalosporins	43 (144/332)	NA	NA	NA
Penicillins	41 (135/332)	NA	NA	NA
Azithromycin	52 (172/332)	NA	NA	NA
Diagnosed with co-infections (e.g., Babesiosis, Bartonellosis, Ehrlichiosis)	90 (321/355)	NA	NA	NA
GSQ-30 total score [mean (SD)]^b^	60 (26)	68 (28)	18 (16)	< 0.01
Impaired functioning in last 2 weeks	85 (321/378)	90 (37/41)	29 (31/108)	< 0.01
Identify as disabled	90 (267/298)	90 (28/31)	41 (39/96)	< 0.01

Includes all individuals who were eligible to participate. Values are percentages (n/N) unless stated otherwise. ^a^*P*-values calculated by Kruskal-Wallis rank sum tests (continuous data) and Fisher’s exact tests (discrete data). ^b^Maximum score on the GSQ-30 is 120 (30 questions, each scored on a 0–4 scale).

Overall, more Diagnosed LD participants had previously tested positive for Lyme disease by standard ELISA and/or Western blot assays (*p* < 0.05) (Table 2). Individuals residing in the United States were more likely to receive their diagnosis in their own country (99%) compared to those living in Canada (78%) or elsewhere (82%) (*p* < 0.01). Almost all Diagnosed LD participants were diagnosed after they exhibited signs of late stage disseminated disease (91%), eventually received treatment specifically for LD (94%), and were further diagnosed with at least one co-infection (90%), most commonly Bartonellosis (65%) and/or Babesiosis (64%) (Table 2). Of those who received antibiotic treatment at some point in their lives, tetracyclines (e.g., doxycycline, minocycline) were most often prescribed (82%) (Table 2). An additional nine Suspected LD participants had also tested positive for Lyme disease by one or more blood test methods (ELISA, Western blot, PCR-DNA, EliSpot, and/or other), despite never receiving a formal diagnosis (Table 2).

### Pregnancy complications and outcomes by participant/parent Lyme disease status

To assess the impact of Lyme disease and LD treatment on gestation and birth outcomes, participants’ pregnancies were classified into one of five groups based on the dates provided in the survey: Probable Treated LD (*n* = 135), Probable Untreated LD (*n* = 124), Possible Untreated LD (*n* = 480), No Evidence of LD (*n* = 364), and Unclear (*n* = 51) pregnancies (Figure 1 and Table 3). This distinction was important as many Diagnosed LD and Suspected LD participants/birthing parents did not develop Lyme disease symptoms until after their pregnancies or, conversely, did not receive a diagnosis or treatment until many years after delivery, if at all, despite being symptomatic before pregnancy. For most pregnancies classified as Probable LD based on a diagnosis date, this diagnosis date was prior to gestation. However, the Probable Treated LD group included eight cases where both diagnosis and treatment were confirmed to occur during the pregnancy, while the Probable Untreated LD group contained one case with a diagnosis during pregnancy, but treatment only commenced after delivery.

**TABLE 3:** Pregnancy classifications.

Probable Treated Lyme Disease pregnancy:
● Parent diagnosed with Lyme disease (LD) before or during the pregnancy
AND/OR
● Onset of an erythema migrans (EM) rash before or during the pregnancy
AND
● Treatment for LD commenced before or during the pregnancy

**Probable Untreated Lyme Disease pregnancy:**
● Parent diagnosed with Lyme disease before or during the pregnancy
AND/OR
● Onset of an EM rash before or during the pregnancy
AND
● No treatment for LD commenced before or during the pregnancy

**Possible Untreated Lyme Disease pregnancy:**
● Parent bit by a tick before or during the pregnancy
AND/OR
● Onset of non-EM Lyme disease symptoms before or during the pregnancy
AND
● No treatment for LD commenced before or during the pregnancy

**No Evidence of Lyme Disease pregnancy:**
● Parent has no diagnosis or suspicion of Lyme disease and no tick bites ever
OR
● Parent has no diagnosis or suspicion of Lyme disease ever and no tick bite prior to or during the pregnancy
OR
● At least two of LD diagnosis, tick bite, EM onset, and non-EM symptom onset dates provided, all of which were after the pregnancy

**Unclear pregnancy:**
● Parent diagnosed with LD after the pregnancy with no other dates available
OR
● At least two of diagnosis, tick bite, EM onset, and non-EM symptom onset dates provided, all after the pregnancy, but the child is noted as diagnosed with congenital Lyme disease
OR
● One or more critical date(s) for classification (including treatment start) is/are the same year as the birth and the month is missing

Pregnancies where the participant showed No Evidence of LD prior to or during gestation experienced significantly fewer complications, such as extreme fatigue (*p* < 0.01), memory issues (*p* < 0.01), fever of unknown origin (*p* < 0.01), exacerbation of joint pain (*p* < 0.01), and postpartum depression (*p* < 0.01), compared to all three LD-affected pregnancy groups, and were most likely to result in a live birth (*p* = 0.01) (Tables 4, 5). However, average parental age at the time of pregnancy was also the lowest in this No Evidence of LD group (*p* < 0.01) (Table 4). Rates of preeclampsia/gestational hypertension and gestational diabetes were similar across all classified pregnancy groups (*p* = 0.43 and *p* = 0.84, respectively) (Table 4). Within the Probable LD groups, obtaining treatment before or during gestation did not impact the risk of developing any of the queried complications (all *p* > 0.12) or affect the pregnancy outcome (*p* = 0.25).

**TABLE 4:** Pregnancy complications and outcomes by participant/parent LD status.

	Probable treated LD pregnancies *n* = 135	Probable untreated LD pregnancies *n* = 124	Possible untreated LD pregnancies *n* = 480	No evidence of LD pregnancies *n* = 364	*P*-value^a^
Parent age at pregnancy, years [mean (SD)]	34 (6)	31 (5)	31 (5)	29 (5)	< 0.01
Any pregnancy complication	82 (108/131)	91 (111/122)	86 (390/453)	53 (180/342)	< 0.01
Vaginal spotting/bleeding	34 (44/131)	39 (48/122)	35 (159/453)	24 (83/342)	< 0.01
Extreme fatigue unresolved by rest	56 (74/131)	59 (72/122)	51 (233/453)	15 (53/342)	< 0.01
Memory issues	44 (58/131)	36 (44/122)	34 (154/453)	8 (27/342)	< 0.01
Exacerbation of joint pain or swelling	37 (49/131)	34 (42/122)	29 (133/453)	6 (20/342)	< 0.01
Bell’s Palsy	4 (5/131)	8 (10/122)	3 (13/453)	0 (0/342)	< 0.01
Hyperemesis gravidarum	16 (21/131)	18 (22/122)	21 (97/453)	11 (36/342)	< 0.01
Irritable uterus	24 (32/131)	24 (29/122)	16 (72/453)	7 (23/342)	< 0.01
Prodromal labor	15 (20/131)	13 (16/122)	16 (72/453)	4 (13/342)	< 0.01
Fever with unknown origin	11 (14/131)	16 (19/122)	11 (50/453)	3 (9/342)	< 0.01
PUPPPs	6 (8/131)	5 (6/122)	4 (16/453)	2 (6/342)	0.07
Preterm labor	9 (12/131)	13 (16/122)	9 (43/453)	6 (19/342)	0.05
Preeclampsia or gestational hypertension	5 (7/131)	7 (9/122)	9 (41/453)	6 (22/342)	0.43
Gestational diabetes	8 (10/131)	7 (8/122)	6 (28/453)	6 (19/342)	0.84
HELLP syndrome	5 (6/131)	1 (1/122)	0 (1/453)	2 (8/342)	< 0.01
Five or more complications	23 (30/131)	20 (24/122)	14 (64/453)	2 (8/342)	< 0.01
Currently pregnant	14 (19/135)	2 (2/124)	0 (1/480)	1 (5/364)	< 0.01
Pregnancy outcomes^b^					0.01
Live birth	68 (79/116)	75 (91/122)	72 (347/479)	79 (282/359)	
Miscarriage	32 (37/116)	25 (30/122)	28 (132/479)	20 (73/359)	
Stillbirth	0 (0/116)	1 (1/122)	0 (0/479)	1 (4/359)	

Only includes pregnancies with a known due date, date of birth, or miscarriage date, and excludes pregnancies with “unclear” timing in relation to diagnosis/treatment/symptoms/tick bite.

Values are percentages (*n*/N) unless stated otherwise. ^a^*P*-values calculated by Kruskal-Wallis rank sum tests (continuous data) and Fisher’s exact tests (discrete data). ^b^Elective abortions were automatically excluded due to missing dates.

**TABLE 5:** Postpartum and newborn outcomes by participant/parent LD status.

	Probable treated LD pregnancies *n* = 79	Probable untreated LD pregnancies *n* = 91	Possible untreated LD pregnancies *n* = 347	No evidence of LD pregnancies *n* = 282	*P*-value^a^
Gestational age at delivery, weeks [mean (SD)]	39 (3)	38 (3)	39 (3)	39 (2)	0.06
Delivery < 34 weeks	4 (3/77)	6 (5/86)	4 (13/318)	2 (6/271)	0.33
Delivery < 37 weeks	18 (14/77)	15 (13/86)	12 (38/318)	13 (35/271)	0.47
Newborn weight, lbs [mean (SD)]	7.41 (1.4)	7.41 (1.4)	7.39 (1.4)	7.63 (1.28)	0.25
Female	43 (34/79)	54 (49/91)	49 (168/346)	50 (141/280)	0.54
Parent postpartum depression	51 (40/79)	43 (39/91)	48 (164/345)	26 (74/281)	< 0.01
Neonatal death	0 (0/79)	0 (0/91)	0 (0/346)	1 (2/279)	0.51
Breastfed	85 (64/75)	90 (78/87)	90 (300/332)	92 (251/273)	0.38
Any signs of pathology in the first 2 weeks of life	43 (32/74)	53 (41/78)	47 (148/315)	33 (87/267)	< 0.01
Hyperbilirubinemia	31 (23/74)	31 (24/78)	35 (111/315)	26 (70/267)	0.14
Adenopathy	0 (0/74)	5 (4/78)	2 (5/315)	1 (2/267)	0.05
Rash	4 (3/74)	15 (12/78)	7 (23/315)	1 (3/267)	< 0.01
Fever of unknown origin	4 (3/74)	10 (8/78)	5 (16/315)	3 (7/267)	0.05
Intrauterine growth restriction	1 (1/74)	1 (1/78)	2 (7/315)	1 (2/267)	0.44
Hypotonia	1 (1/74)	9 (7/78)	1 (2/315)	1 (2/267)	< 0.01
Respiratory distress	9 (7/74)	24 (19/78)	10 (33/315)	5 (14/267)	< 0.01
Any congenital anomaly	9 (7/75)	7 (6/84)	8 (27/327)	8 (21/272)	0.95
Syndactyly	14 (1/7)	17 (1/6)	7 (2/27)	5 (1/21)	0.46
Heart defect	14 (1/7)	50 (3/6)	30 (8/27)	19 (4/21)	0.45
Urologic/urogenital defect	14 (1/7)	17 (1/6)	7 (2/27)	0 (0/21)	0.20
Cleft lip and/or palate	0 (0/7)	0 (0/6)	4 (1/27)	0 (0/21)	1
Neural tube defect	0 (0/7)	0 (0/6)	7 (2/27)	0 (0/21)	0.69
Other	57 (4/7)	50 (3/6)	67 (18/27)	81 (17/21)	0.37

Only includes live births with a known date of birth and excludes pregnancies with “unclear” timing in relation to diagnosis/treatment/symptoms/tick bite. Values are percentages (n/N) unless stated otherwise. ^a^*P*-values calculated by Kruskal-Wallis rank sum tests (continuous data) and Fisher’s exact tests (discrete data).

Testing of fetal-associated tissues (cord tissue, cord blood, placental tissue, fetal tissue, or foreskin) after birth or miscarriage for evidence of gestational transmission of *Borrelia burgdorferi* was reported following 28 pregnancies (25 Probable LD pregnancies and three that did not provide a DOB for classification), mostly involving United States residents (76%). These 25 Probable LD pregnancies represented only 17% of all classified pregnancies where the participant had a known diagnosis of Lyme disease before the delivery or miscarriage (*n* = 149) and only 3% of all classified pregnancies where vertical transmission was possible (Probable or Possible LD; *n* = 739). Positive or equivocal (borderline/inconclusive) results were obtained in four out of 28 cases (14%; all cord blood samples, plus one placenta sample that was also positive). In one of the two positive cases, the birthing parent received oral antibiotics for Lyme disease around the third month of gestation, while in the other, the participant was treated with multiple antibiotics in the same year as the DOB, but it was unclear if this was during or after the pregnancy. In both cases with equivocal cord blood results, the participant received more than 4 weeks of treatment several years before the pregnancy but experienced a recurrence of symptoms prior to conception. All cases with confirmed negative results, in which the timing of treatment relative to pregnancy was provided, had also been treated with antibiotics before or during gestation.

### Child outcomes by participant/parent Lyme disease status

Pregnancies that resulted in a live birth before the parent participated in the survey (73%, *n* = 845) were further investigated to examine associations between the birthing parents’ Lyme disease/treatment status and neonatal/child health outcomes (Figure 1). In the first 2 weeks of life, newborns from Probable Untreated LD pregnancies were most likely to experience rashes (*p* < 0.01), unexplained fevers (*p* = 0.05), hypotonia (*p* < 0.01), and respiratory distress (*p* < 0.01), compared to Probable Treated LD, Possible Untreated LD, and No Evidence of LD offspring (Table 5). The frequency of congenital anomalies was similar across all groups (all *p* > 0.20) (Table 5).

When offspring of all ages were included in the analysis of long-term outcomes, those from Untreated LD pregnancies (Probable and Possible) demonstrated the highest rates of musculoskeletal, gastrointestinal/urinary, sleep, concentration/fatigue, sensory, and vision issues, along with recurrent infections and dizziness (all *p* < 0.01) (Table 6). Offspring from Untreated LD parents were also most likely to eventually be diagnosed with Lyme disease themselves (*p* < 0.01), along with a wide range of other diagnoses, including allergy/immune, orthopedic, cardiovascular/respiratory, neurological, gastrointestinal, dermatologic, endocrine, genitourinary/renal, ocular, and mental health disorders (all *p* < 0.01) (Table 6). Children from Treated LD pregnancies appeared to fare significantly better than those from Untreated LD pregnancies, with LD symptoms, health issues, and rates of medical diagnoses comparable to No Evidence of LD children (Table 6). However, these Probable Treated LD offspring were the youngest at the time of their parents’ participation in the survey [mean of 5 years old and maximum of 29 years old, compared to a mean of 13–16 years old and maximum of 48–54 years old in the other three groups (*p* < 0.01)] (Table 6), so health issues associated with later childhood/adolescence would be less likely to have manifested in this group.

**TABLE 6:** Child outcomes by participant/parent LD status.

	Probable treated LD pregnancies *n* = 79	Probable untreated LD pregnancies *n* = 91	Possible untreated LD pregnancies *n* = 347	No evidence of LD pregnancies *n* = 282	*P*-value^a^
Child age at parent participation, years [mean (SD)]	5 (6)	16 (11)	13 (9)	14 (10)	< 0.01
Diagnosed with Lyme disease	19 (15/78)	42 (38/90)	44 (150/342)	14 (40/279)	< 0.01
Congenital Lyme disease	73 (11/15)	61 (22/36)	59 (89/150)	0 (0/40)	< 0.01
Child age at LD diagnosis, years [mean (SD)]	3 (2)	12 (10)	10 (6)	12 (6)	< 0.01
Suspected Lyme disease	28 (22/78)	26 (23/90)	30 (103/341)	10 (28/278)	< 0.01
Any tick bite	21 (16/78)	17 (15/89)	20 (69/339)	16 (45/279)	0.53
Any musculoskeletal symptoms	40 (29/73)	64 (54/84)	61 (194/318)	26 (71/269)	< 0.01
Any gastrointestinal/urinary symptoms	65 (46/71)	79 (67/85)	76 (244/319)	40 (106/262)	< 0.01
Any recurrent infections	43 (30/70)	74 (63/85)	66 (212/319)	40 (104/263)	< 0.01
Any non-specific symptoms	74 (55/74)	87 (74/85)	85 (278/326)	52 (139/269)	< 0.01
Night sweats	15 (11/74)	26 (22/85)	24 (79/326)	9 (23/269)	< 0.01
Excessive sweating	12 (9/74)	19 (16/85)	15 (50/326)	6 (15/269)	< 0.01
Sleep issues	35 (26/74)	52 (44/85)	47 (153/326)	21 (56/269)	< 0.01
General fatigue	27 (20/74)	48 (41/85)	47 (153/326)	19 (50/269)	< 0.01
Difficulty concentrating	24 (18/74)	51 (43/85)	53 (172/326)	19 (50/269)	< 0.01
“Brain fog”	16 (12/74)	35 (30/85)	38 (123/326)	12 (32/269)	< 0.01
Limb weakness	7 (5/74)	24 (20/85)	15 (49/326)	7 (19/269)	< 0.01
Dizziness	7 (5/74)	29 (25/85)	23 (76/326)	10 (26/269)	< 0.01
Tingling/numbness	11 (8/74)	29 (25/85)	18 (58/326)	9 (25/269)	< 0.01
Palpitations	7 (5/74)	16 (14/85)	17 (57/326)	8 (22/269)	< 0.01
Sensory issues	39 (29/74)	45 (38/85)	47 (152/326)	16 (42/269)	< 0.01
Vision issues	14 (10/74)	24 (20/85)	20 (64/326)	9 (25/269)	< 0.01
Colic	14 (10/74)	24 (20/85)	25 (82/326)	13 (34/269)	< 0.01
Failure to thrive	3 (2/74)	11 (9/85)	11 (35/326)	2 (6/269)	< 0.01
Hair loss/bald spots	5 (4/74)	12 (10/85)	6 (18/326)	3 (8/269)	0.02
Severe diaper rashes	15 (11/74)	22 (19/85)	18 (58/326)	6 (17/269)	< 0.01
Rashes or skin lesions	22 (16/74)	26 (22/85)	22 (73/326)	10 (28/269)	< 0.01
Fevers of unknown origin	12 (9/74)	16 (14/85)	16 (52/326)	3 (9/269)	< 0.01
Any allergy/immunology/hematologic diagnosis	33 (24/72)	54 (44/82)	51 (157/306)	34 (90/262)	< 0.01
Any orthopedic/rheumatologic diagnosis	13 (9/67)	40 (29/73)	32 (93/292)	15 (36/242)	< 0.01
Any cardiovascular/respiratory diagnosis	16 (11/68)	46 (33/72)	32 (92/284)	18 (44/247)	< 0.01
Any functional/psychosomatic/pain diagnosis	16 (11/67)	30 (21/69)	31 (87/280)	9 (21/246)	< 0.01
Any neurological diagnosis	15 (10/67)	39 (29/74)	42 (121/287)	16 (40/250)	< 0.01
Any gastrointestinal diagnosis	21 (14/68)	41 (29/71)	41 (116/285)	16 (41/251)	< 0.01
Any dermatologic diagnosis	36 (25/69)	57 (42/74)	48 (142/295)	23 (58/251)	< 0.01
Any endocrine diagnosis	4 (3/67)	20 (14/71)	14 (39/277)	8 (20/249)	< 0.01
Any genitourinary/renal diagnosis	8 (5/66)	23 (17/74)	18 (49/268)	11 (28/250)	< 0.01
Any ocular diagnosis	9 (6/64)	23 (17/73)	15 (41/272)	7 (17/242)	< 0.01
Any mental health/developmental diagnosis	45 (33/73)	76 (65/85)	70 (221/316)	46 (120/262)	< 0.01
Other diagnosis	10 (6/63)	32 (24/74)	27 (72/267)	12 (27/229)	< 0.01
Any adverse vaccine reaction	14 (10/74)	22 (19/85)	27 (87/328)	11 (31/271)	< 0.01

Includes all live births with a known date of birth and excludes pregnancies with “unclear” timing in relation to diagnosis/treatment/symptoms/tick bite. Values are percentages (n/N) unless stated otherwise. ^a^*P*-values calculated by Kruskal-Wallis rank sum tests (continuous data) and Fisher’s exact tests (discrete data).

A weak correlation was confirmed between child age and the overall number of symptoms (tau = 0.22, *p* < 0.01) or medical diagnoses (tau = 0.31, *p* < 0.01) noted by their parent (Supplementary Figure 1). Therefore, to reduce the confounding effect of child age on health outcomes, the data were re-analyzed with subsets of similarly aged children. When restricted to offspring 6–15 years old at the time of the study [44% of all children (29–51% of each group); *p* = 0.08 for age], those from Probable Treated LD pregnancies demonstrated musculoskeletal, gastrointestinal/urinary, sleep, concentration/fatigue, sensory, and vision issues, as well as rashes and unexplained fevers, at similarly elevated rates to children from Probable and Possible Untreated LD pregnancies (Table 7). Orthopedic, cardiovascular/respiratory, gastrointestinal, dermatologic, and Lyme disease diagnoses were also prevalent in all three LD-affected groups, compared to No Evidence of LD children (all *p* < 0.01) (Table 7). However, some pathologies were still most frequently observed in Probable (and sometimes Possible) Untreated LD 6–15-year-olds, such as recurrent infections (*p* < 0.01), limb weakness (*p* = 0.01), dizziness (*p* = 0.01), allergy/immune diagnoses (*p* = 0.02), and neurological diagnoses (*p* < 0.01) (Table 7). A similar pattern was identified in the smaller and/or less balanced subsets of 2–8-year-olds [30% of all children (18–59% of each group); *p* = 0.09 for age] (Supplementary Table 2) and 9–22-year-olds [48% of all children (16–55% of each group); *p* = 0.06 for age] (Supplementary Table 3).

**TABLE 7:** Outcomes in children between the ages of six and 15 by participant/parent LD status.

	Probable treated LD pregnancies *n* = 23	Probable untreated LD pregnancies *n* = 33	Possible untreated LD pregnancies *n* = 178	No evidence of LD pregnancies *n* = 113	*P*-value^a^
Child age at parent participation, years [mean (SD)]	9 (3)	11 (3)	10 (3)	10 (3)	0.08
Diagnosed with Lyme disease	48 (11/23)	48 (16/33)	45 (80/176)	14 (16/113)	< 0.01
Congenital Lyme disease	82 (9/11)	88 (14/16)	56 (45/80)	0 (0/16)	< 0.01
Child age at LD diagnosis, years [mean (SD)]	3 (2)	7 (3)	7 (3)	7 (3)	< 0.01
Suspected Lyme disease	22 (5/23)	27 (9/33)	30 (53/175)	16 (18/113)	0.04
Any tick bite	30 (7/23)	16 (5/32)	23 (40/173)	18 (20/113)	0.40
Any musculoskeletal symptoms	68 (15/22)	73 (22/30)	65 (108/165)	27 (30/110)	< 0.01
Any gastrointestinal/urinary symptoms	86 (18/21)	87 (26/30)	82 (134/164)	44 (47/107)	< 0.01
Any recurrent infections	43 (9/21)	80 (24/30)	65 (106/164)	42 (44/105)	< 0.01
Any non-specific symptoms	91 (20/22)	90 (28/31)	88 (148/168)	52 (57/110)	< 0.01
Night sweats	18 (4/22)	29 (9/31)	26 (43/168)	9 (10/110)	< 0.01
Excessive sweating	9 (2/22)	19 (6/31)	12 (21/168)	5 (5/110)	0.04
Sleep issues	50 (11/22)	58 (18/31)	50 (84/168)	24 (26/110)	< 0.01
General fatigue	59 (13/22)	52 (16/31)	45 (75/168)	15 (16/110)	< 0.01
Difficulty concentrating	55 (12/22)	58 (18/31)	50 (84/168)	18 (20/110)	< 0.01
“Brain fog”	36 (8/22)	35 (11/31)	35 (59/168)	8 (9/110)	< 0.01
Limb weakness	9 (2/22)	32 (10/31)	14 (24/168)	8 (9/110)	0.01
Dizziness	14 (3/22)	32 (10/31)	22 (37/168)	10 (11/110)	0.01
Tingling/numbness	23 (5/22)	32 (10/31)	15 (25/168)	9 (10/110)	0.01
Palpitations	18 (4/22)	16 (5/31)	15 (26/168)	6 (7/110)	0.07
Sensory issues	68 (15/22)	55 (17/31)	51 (85/168)	20 (22/110)	< 0.01
Vision issues	27 (6/22)	35 (11/31)	21 (36/168)	9 (10/110)	< 0.01
Colic	5 (1/22)	13 (4/31)	21 (35/168)	14 (15/110)	0.17
Failure to thrive	5 (1/22)	10 (3/31)	10 (16/168)	2 (2/110)	0.04
Hair loss/bald spots	5 (1/22)	19 (6/31)	1 (2/168)	3 (3/110)	< 0.01
Severe diaper rashes	14 (3/22)	26 (8/31)	16 (27/168)	9 (10/110)	0.10
Rashes or skin lesions	41 (9/22)	32 (10/31)	24 (40/168)	9 (10/110)	< 0.01
Fevers of unknown origin	18 (4/22)	19 (6/31)	18 (30/168)	3 (3/110)	< 0.01
Any allergy/immunology/hematologic diagnosis	45 (10/22)	65 (20/31)	52 (82/158)	36 (38/105)	0.02
Any orthopedic/rheumatologic diagnosis	20 (4/20)	33 (9/27)	27 (40/149)	8 (8/96)	< 0.01
Any cardiovascular/respiratory diagnosis	35 (7/20)	42 (11/26)	31 (46/147)	13 (13/97)	< 0.01
Any functional/psychosomatic/pain diagnosis	42 (8/19)	31 (8/26)	23 (32/139)	3 (3/97)	< 0.01
Any neurological diagnosis	21 (4/19)	46 (13/28)	43 (64/150)	13 (13/99)	< 0.01
Any gastrointestinal diagnosis	35 (7/20)	41 (11/27)	36 (53/147)	13 (13/101)	< 0.01
Any dermatologic diagnosis	55 (11/20)	56 (15/27)	47 (72/154)	26 (27/104)	< 0.01
Any endocrine diagnosis	11 (2/19)	16 (4/25)	10 (15/143)	4 (4/100)	0.10
Any genitourinary/renal diagnosis	16 (3/19)	23 (6/26)	14 (20/142)	10 (10/100)	0.33
Any ocular diagnosis	24 (4/17)	20 (5/25)	14 (20/143)	7 (7/101)	0.07
Any mental health/developmental diagnosis	67 (14/21)	81 (25/31)	72 (118/164)	51 (55/107)	< 0.01
Other diagnosis	12 (2/17)	44 (12/27)	32 (46/144)	11 (10/93)	< 0.01
Any adverse vaccine reaction	23 (5/22)	19 (6/31)	30 (51/169)	15 (16/110)	0.02

Only includes live births with a known date of birth, where the child was between 6 and 15 years old when their parent participated in the survey, and excludes pregnancies with “unclear” timing in relation to diagnosis/treatment/symptoms/tick bite. Values are percentages (n/N) unless stated otherwise. ^a^*P*-values calculated by Kruskal-Wallis rank sum tests (continuous data) and Fisher’s exact tests (discrete data).

### Child outcomes by child Lyme disease status

Lyme disease had been diagnosed in 33% of children (*n* = 279) with a known DOB and LD status at the time of their parents’ participation in the survey (Figure 1 and Table 8). While 54% of these infections were noted as being congenital in origin, this was impossible to confirm given the retrospective nature of the survey. However, only 29% of Diagnosed LD offspring had a known tick bite, and only 12% had ever shown an EM rash, which might suggest an alternative infection source (Table 8). Children were diagnosed with Lyme disease at an average age of 11 years old, were tested mainly using standard ELISA and/or Western blot assays, and 84% were also positive for at least one co-infection (Table 8). An additional 180 children (22%) were suspected of having Lyme disease, while the remaining 376 (45%) had never been diagnosed, nor did their parent (the participant) suspect they ever had LD (Figure 1). When these three groups were compared, Diagnosed LD offspring were consistently associated with the highest rates of Lyme disease symptoms and comorbidities, while outcomes in Suspected LD children were also more severe than those observed in the No LD group (all *p* < 0.01) (Table 8). However, there was once again a significant difference in the average age of the children across these three LD groups (*p* < 0.01) (Table 8 and Supplementary Figure 1).

**TABLE 8:** Child outcomes by child LD status.

	Child diagnosed LD *n* = 279	Child suspected LD *n* = 180	Child no diagnosis or suspicion *n* = 376	*P*-value^a^
Parent Lyme disease status during pregnancy				<0.01
Probable treated Lyme disease	6 (15/243)	12 (22/176)	11 (41/370)	
Probable untreated Lyme disease	16 (38/243)	13 (23/176)	8 (29/370)	
Possible untreated Lyme disease	62 (150/243)	59 (103/176)	24 (89/370)	
No evidence of Lyme disease	16 (40/243)	16 (28/176)	57 (211/370)	
Child age at parent participation, years [mean (SD)]	15 (9)	13 (9)	12 (11)	<0.01
Child age at LD diagnosis, years [mean (SD)]	11 (7)	NA	NA	NA
Any tick bite	29 (79/275)	20 (36/180)	12 (44/376)	<0.01
Erythema migrans (EM) rash	12 (33/267)	19 (7/36)	2 (1/44)	0.04
Flu-like symptoms of Lyme disease	64 (171/268)	NA	NA	NA
Cardiac symptoms of Lyme disease	13 (36/268)	NA	NA	NA
Neurological symptoms of Lyme disease	25 (67/268)	NA	NA	NA
Joint symptoms of Lyme disease	42 (112/268)	NA	NA	NA
Ocular symptoms of Lyme disease	15 (39/268)	NA	NA	NA
Borrelial lymphocytoma	1 (4/268)	NA	NA	NA
Other Lyme disease symptoms	47 (125/268)	NA	NA	NA
Positive ELISA or western blot	36 (73/203)	NA	NA	NA
Positive ELISA and western blot	38 (78/203)	NA	NA	NA
Positive other blood test	36 (74/203)	NA	NA	NA
Positive non-blood Lyme disease test	30 (83/277)	NA	NA	NA
Diagnosed with co-infections (e.g., babesiosis, bartonellosis, ehrlichiosis)	84 (194/231)	NA	NA	NA
Any musculoskeletal symptoms	80 (208/261)	60 (100/167)	19 (68/360)	<0.01
Any gastrointestinal/urinary symptoms	88 (228/259)	81 (137/169)	37 (130/353)	<0.01
Any recurrent infections	74 (191/259)	68 (112/164)	38 (136/356)	<0.01
Any non-specific symptoms	99 (263/267)	90 (153/170)	47 (171/362)	<0.01
Night sweats	41 (109/267)	18 (31/170)	3 (10/362)	<0.01
Excessive sweating	22 (58/267)	14 (23/170)	5 (17/362)	<0.01
Sleep issues	60 (160/267)	49 (83/170)	15 (54/362)	<0.01
General fatigue	67 (180/267)	48 (81/170)	7 (26/362)	<0.01
Difficulty concentrating	69 (185/267)	45 (76/170)	13 (47/362)	<0.01
“Brain fog”	58 (154/267)	25 (43/170)	5 (19/362)	<0.01
Limb weakness	28 (75/267)	14 (24/170)	2 (7/362)	<0.01
Dizziness	40 (108/267)	18 (30/170)	2 (7/362)	<0.01
Tingling/numbness	36 (97/267)	12 (21/170)	2 (6/362)	<0.01
Palpitations	29 (78/267)	15 (26/170)	2 (6/362)	<0.01
Sensory issues	63 (169/267)	41 (69/170)	12 (44/362)	<0.01
Vision issues	33 (87/267)	16 (27/170)	5 (17/362)	<0.01
Colic	24 (64/267)	22 (38/170)	13 (47/362)	<0.01
Failure to thrive	11 (30/267)	9 (15/170)	4 (13/362)	<0.01
Hair loss/bald spots	8 (21/267)	9 (15/170)	2 (6/362)	<0.01
Severe diaper rashes	19 (51/267)	18 (31/170)	8 (30/362)	<0.01
Rashes or skin lesions	31 (84/267)	26 (44/170)	6 (23/362)	<0.01
Fevers of unknown origin	21 (57/267)	16 (27/170)	2 (7/362)	<0.01
Any allergy/immunology/hematologic diagnosis	64 (161/251)	51 (80/157)	28 (101/357)	<0.01
Any orthopedic/rheumatologic diagnosis	41 (93/226)	33 (47/144)	11 (39/341)	<0.01
Any cardiovascular/respiratory diagnosis	47 (107/228)	28 (39/138)	15 (51/345)	<0.01
Any functional/psychosomatic/pain diagnosis	46 (97/213)	29 (41/141)	5 (18/345)	<0.01
Any neurological diagnosis	53 (117/222)	43 (63/148)	11 (38/347)	<0.01
Any gastrointestinal diagnosis	52 (117/223)	36 (51/142)	13 (46/349)	<0.01
Any dermatologic diagnosis	53 (120/228)	52 (77/148)	25 (88/353)	<0.01
Any endocrine diagnosis	29 (63/219)	7 (10/139)	4 (14/347)	<0.01
Any genitourinary/renal diagnosis	29 (62/212)	17 (22/133)	8 (27/350)	<0.01
Any ocular diagnosis	20 (40/202)	14 (20/143)	8 (27/341)	<0.01
Any mental health/developmental diagnosis	82 (213/260)	73 (121/165)	39 (140/357)	<0.01
Other diagnosis	37 (75/201)	30 (40/132)	7 (25/334)	<0.01
Any adverse vaccine reaction	33 (87/265)	25 (43/171)	9 (34/368)	<0.01

Only includes live births with a known date of birth. Values are percentages (*n*/N) unless stated otherwise. ^a^*P*-values calculated by Kruskal-Wallis rank sum tests (continuous data) and Fisher“2019”s exact tests (discrete data).

## Discussion

Our survey is the largest survey ever conducted focused on Lyme disease in pregnancy. It represents an important step toward addressing current research gaps regarding the impact of LD on gestational complications and neonatal/child outcomes, as well as the risk of perinatal transmission. Overall, we found that both participants and their children who had been diagnosed with, or suspected that they have, Lyme disease reported managing a high symptom burden and several co-morbidities. In this study, treatment for LD was not associated with decreased pregnancy complications or improved birth outcomes but was associated with reduced rates of select neonatal pathology (e.g., rashes, hypotonia, and respiratory distress). Our findings additionally suggest that there are substantial and non-specific long-term effects of LD exposure *in utero* on child health, which may be somewhat attenuated by parental treatment for LD. Unfortunately, confirmation of congenital infection was unavailable in most cases, as testing of fetal/newborn tissues after birth or miscarriage occurred for only 3% of pregnancies at risk of transmission.

A concerning finding from this study was the severity of symptoms in people with Suspected LD, as well as the frequency of positive Lyme disease tests in this group, despite never receiving an official diagnosis by a medical professional. This Suspected LD group was also associated with the lowest attained education levels and household incomes, indicating that financial hardship, employment status, and/or a lack of insurance (depending on their country of residence) may be affecting their access to health care, delaying diagnosis and treatment, and negatively influencing outcomes (48). Many additional barriers preventing diagnosis in this group are also possible (1, 48). When pregnancies were classified based on the likelihood of *Borrelia burgdorferi* infection at the time of gestation, participants with Possible or Probable LD (including those with undiagnosed but Suspected LD) experienced much higher rates of pregnancy complications, such as extreme fatigue, joint pain, fever of unknown origin, and postpartum depression, compared to those with No Evidence of LD. These symptoms are all potentially consistent with a Lyme disease diagnosis (1, 5, 49) and are not necessarily a function of the pregnancy itself (31), although a comparison of symptoms before and during pregnancy in the same cohort of people would be of interest. LD-affected pregnancies were also associated with a higher rate of miscarriage, supporting an association between Lyme disease and spontaneous abortion, as previously documented (30, 31, 33). No significant differences in stillbirth or neonatal death rates were observed; however, these outcomes were rare (*n* = 5 stillbirths and *n* = 2 neonatal deaths).

Children born from pregnancies classified as Possible or Probable LD demonstrated a wide range of pathologies, including musculoskeletal, gastrointestinal, orthopedic, cardiovascular, respiratory, sleep, rashes, fevers, concentration, sensory, and vision issues. This is consistent with previous research (24, 30, 32), which has been unable to identify a specific syndrome associated with potential congenital *Borrelia burgdorferi* infection in offspring. However, unlike prior studies, we did not observe any significant differences between children from LD-affected pregnancies and No Evidence of LD pregnancies in terms of gestational age at delivery, intrauterine growth restriction diagnoses, congenital anomalies, or hyperbilirubinemia rates (24, 31, 32). Unfortunately, given the lack of *Borrelia burgdorferi* testing done on fetal/neonatal tissues, we cannot determine how many of the reported child outcomes were truly due to the transmission of the bacteria itself or were, instead, caused by other factors. Possible other contributors include an independent Lyme disease exposure in the child, parental or child co-infections (50, 51), a genetic predisposition to infectious or inflammatory conditions (52), and the overall impact of a parental pathological state in pregnancy on child growth and development (53–56).

The effect of birthing parent treatment for Lyme disease on offspring health outcomes was also challenging to disassociate from a confounding factor: child age at the time of parent participation in the survey. In general, we discovered a weak correlation between offspring age and the total number of LD-associated symptoms or medical diagnoses noted by their parent. Children from Treated Probable LD pregnancies were also significantly younger than those from Untreated or No Evidence of LD groups, which might indicate that timely diagnosis and subsequent treatment for Lyme disease has become more prevalent in recent years. These findings further confirm the importance of longitudinal follow-up in offspring with potential perinatal LD exposure. When this age discrepancy was appropriately considered, our results support the hypothesis that parent treatment for Lyme disease before or during pregnancy can attenuate severe pathology in neonates/children but does not eliminate poor outcomes entirely (30, 32). In this study, offspring with treated parents revealed reduced rates of neonatal rashes, fever, hypotonia, and respiratory distress. Nevertheless, over time, they developed many of the same pathologies as their counterparts with untreated parents but were still less likely to experience specific issues, such as recurrent infections, limb weakness, and dizziness. Further studies are warranted and urgently needed to compare (age-matched) long-term child outcomes from treated and untreated LD pregnancies.

### Study limitations

While this research represents substantial progress toward understanding the possible effects of Lyme disease on pregnancy and offspring health, it is not without limitations. Since recruitment was primarily through North American-based Lyme disease advocacy groups, participants were more likely to have experienced chronic/late-stage disease with persistent symptoms than would be expected from a random group of LD patients (7, 57). Most participants also resided in Canada or the United States, despite international eligibility. As such, our findings may not extend to other Lyme disease populations. Additionally, as a cross-sectional survey, the accuracy of our results is dependent on the accuracy of the participant-provided information, including the LD-associated dates used for pregnancy classification. Given that many of these dates were 10–70 years prior to participation, we expected some discrepancies due to recall issues. This concern was mitigated during the data cleaning process by identifying and removing biologically impossible data points. Finally, another consequence of the data’s highly variable and self-reported nature was the necessity to group the participants very broadly, despite known differences that may have important biological effects. For example, LD diagnosed with varying criteria and tests, *Borrelia burgdorferi* infection before vs. during pregnancy, treatments with different efficacies, and treated vs. resolved Lyme disease were not distinguished (1, 7, 33, 57). Significant differences were also observed between the study groups in several baseline characteristics, such as participant location, household income, and parent age at the time of pregnancy (1, 48, 58, 59). It is essential that these variables are thoroughly considered in future studies employing more reliable data collection methods.

## Conclusion

Overall, this survey provides an important foundation upon which hypotheses can be generated for many overdue projects focused on Lyme disease in pregnancy. Our results provide evidence of the need for rigorous prospective observational studies that can minimize bias in assessing the impact of LD on pregnancy outcomes and health outcomes of offspring exposed to LD *in utero*. Research priorities include consistent testing of fetal/neonatal tissues at birth for *Borrelia* (and other tick-vectored pathogens) to determine the true frequency of transmission, as well as to help distinguish between the various factors that may contribute to similar symptoms in children (1, 60). Hopefully, future research will lead to evidence-based clinical guidance and resources for healthcare providers, allowing for improved and prompt diagnosis, treatment, and care for both parents and children with Lyme disease.

## Data availability statement

The datasets presented in this article are not readily available because consent to share the data beyond the research team was not collected from the research participants. Requests to access the datasets should be directed to the corresponding author.

## Ethics statement

The studies involving human participants were reviewed and approved by the Hamilton Integrated Research Ethics Board (HiREB) (project #11222). The patients/participants provided their written informed consent to participate in this study.

## Author contributions

RM, SF, VL, CM, MW, IB, EC, AO, OM, and ED conceived and designed the survey. RM, SF, OM, and ED developed and disseminated the recruitment materials. KL and RM performed the data analysis. KL wrote the first draft of the manuscript. All authors contributed to manuscript revision, read, and approved the submitted version.
