# Supplementary material for: Lyme borreliosis in pregnancy and associations with parent and offspring health outcomes: An international cross-sectional survey
Source: Front Med (Lausanne). 2022 Nov 3;9:1022766. doi: 10.3389/fmed.2022.1022766 (PMC9669415; doi:10.3389/fmed.2022.1022766)
Supplement: Supplementary file 1 [file Table_1.PDF]

Supplementary Table 1. Survey questions.

Questions	Responses
1. Have you ever been pregnant?	1, Yes<br>0, No<br>*[If NO, end survey]*

SECTION 1: DIAGNOSIS AND TREATMENT OF LYME DISEASE

Questions	Responses
2. Has a medical professional ever diagnosed you with Lyme Disease?	1, Yes<br>0, No<br>*[If NO, skip to SECTION 2]*
To be completed by participants who have been diagnosed with Lyme Disease	
3. Who made the final Lyme Disease diagnosis?	1, My family doctor<br>2, An emergency physician (walk in clinic, hospital, urgent care, etc.)<br>3, A specialist (please specify)<br>4, A physical therapist<br>5, Naturopath<br>6, Homeopath<br>7, Alternative healer<br>8, Other (please specify)
4. When were you diagnosed with Lyme Disease?	MM/YYYY
5. Where were you diagnosed with Lyme Disease?	1, In my own state/province<br>2, In another state/province in my own country<br>3, In another country
6. How were you diagnosed with Lyme Disease? *Check all that apply*	1, Clinical presentation of erythema migrans (EM) rash<br>2, Clinical diagnosis based on other (non-EM) symptoms/signs<br>3, Blood test (please specify)<br>4, Urine test<br>5, Joint tap<br>6, Cerebrospinal fluid (“spinal tap”)<br>7, Response to antibiotic therapy<br>8, Other (please specify)<br>55, I don’t know or can’t remember

7. *[If diagnosis was via blood test]* Which blood test was performed? *Check all that apply*	1, ELISA / EIA (IgM/IgG)<br>2, Western Blot or immunoblot (IgM/IgG)<br>3, Two-tiered serology (ELISA / EIA + reflex Western blot)<br>4, PCR-DNA<br>5, T-cell response (EliSpot)<br>6, Other (Please specify)<br>55, I don't know or can't remember
8. Did you have a tick removed from your body at any time *prior* to being diagnosed with Lyme Disease?	1, Yes<br>0, No<br>55, I don't know or can't remember<br>*[If No or Can't remember, skip to 18]*
9. How many times did you have a tick removed from your body *prior* to being diagnosed with Lyme Disease?	##
Tick Module: For each tick removed, answer questions 10 - 16	
10. *[If yes to tick being removed]* Did you acquire this tick in a region where Lyme Disease is endemic (very commonly found)?	1, Yes<br>0, No55, I don't know or can't remember
11. *[If yes to tick being removed]* Approximately when was the tick removed?	MM/YYYY
12. *[If yes to tick being removed]* Was the removed tick submitted for testing for Lyme Disease?	1, Yes<br>0, No<br>55, I don't know or can't remember
13. *[If yes to tick being submitted]* Was the species of tick identified?	1, Yes<br>0, No<br>2, Unknown (did not receive results)<br>55, Can't remember
14. *[If yes to tick being submitted]* What was the species of tick that was identified?	1, Lone star tick (*Amblyomma americanum)*<br>2, Black legged tick (*Ixodes scapularis)*<br>3, Western black-legged tick (*Ixodes pacificus)*<br>4, *Ixodes angustus*<br>5, Castor bean tick (*Ixodes ricinus)*<br>6, Woodchuck / groundhog tick *(Ixodes cookei)*<br>7, Brown dog tick (*Rhipicephalus sanguineus*)<br>8, American dog tick (*Dermacentor variablis*)<br>9, Gulf coast tick (*Amblyomma maculatum*)<br>10, Other (please specify)<br>11, Unknown (did not receive results)<br>55, Can't remember

15. *[If yes to tick being submitted]* Did the tick test positive for *Borrelia burgdorferi* (main bacterium that causes Lyme Disease in North America)?	1, Yes<br>0, No<br>2, Unknown (did not receive results)<br>3, Lab refused to test<br>55, Can't remember
16. *[If yes to tick being submitted]* Did the tick test positive for any of the following bacteria? *Check all that apply*	1, *Anaplasma* spp.<br>2, *Babesia* spp.<br>3, *Bartonella* spp.<br>*4, Borrelia afzelii*<br>*5, Borrelia garinii*<br>6, *Borrelia mayonii*<br>*7, Borrelia bissetti*<br>8, Relapsing fever *Borrelia* (incl. *B. hermsii, B. perkerii, B. turicatae, B. miyamotoi, B. Lonestari*)<br>9, *Ehrlichia* spp.<br>10, *Rickettsia* spp.<br>11, Other (please specify)<br>12, Unknown (did not receive results)<br>13, Tick was not tested for these bacteria<br>55, Can't remember
17. Did you develop an erythema migrans rash (sometimes called a "bullseye rash") prior to your Lyme Disease diagnosis? [An erythema migrans rash is a red, expanding bull's-eye looking rash or a circular/oval rash on an area of skin. On darker skin, the rash may have the appearance of a bruise, rather than being red, but would still be enlarging.]	1, Yes<br>0, No<br>55, I don't know or can't remember
18. *[If yes to EM rash]* Approximately when did the rash develop?	MM/YYYY
19. Prior to your diagnosis, did you experience any of the following symptoms of Lyme Disease? Check all that apply	1, Fever<br>2, Recurring headaches<br>3, Recurring neck pain<br>4, Swelling of joints<br>5, Joint pain<br>6, Muscle pain<br>7, Fatigue<br>8, Facial drooping<br>9, Numbness and tingling<br>10, Muscle spasms or twitches<br>11, Dysfunction of the brain (including word retrieval and memory issues)<br>12, Lack of joint/muscle stability or function<br>13, Other Lyme Disease symptoms not listed (please specify)

	44, None of the above<br>55, Can't remember
20. *[If yes to any LD symptoms]* Approximately when did your Lyme Disease symptoms start?	MM/YYYY
21. Have you ever received treatment specifically for Lyme Disease?	1, Yes<br>0, No<br>55, I don't know or can't remember<br>*[If No or Can't remember, skip to SECTION 3]*
22. When did you first receive treatment for Lyme Disease?	MM/YYYY
23. What treatment(s) have you received for Lyme Disease? *Check all that apply*	1, Oral antibiotics<br>2, Intravenous antibiotics<br>3, Other (please specify)<br>55, I don't know or can't Remember
24. *[If yes to antibiotic treatment]* Which antibiotic was prescribed? *Check all that apply*	1, Tetracyclines (e.g. doxycycline, minocycline)<br>2, Cephalosporins (e.g. ceftriaxone, cefuroxime)<br>3, Penicillins (e.g. amoxicillin)<br>4, Azithromycin<br>5, Other (please specify)<br>55, I don't know or can't remember
25. *[If yes to antibiotic treatment]* For how long were you prescribed antibiotics? *Check all that apply*	1, Single dose / one day (e.g. tick bite prophylaxis)<br>2, 10-21 days<br>3, 28 days<br>4, Other (please specify)<br>55, I don't know or can't remember
26. *[If yes to single or standard dose antibiotic treatment]* Did you take the complete course of antibiotics?	1, Yes<br>0, No<br>55, I don't know or can't remember
27. *[If yes to antibiotic treatment]* What was your response to antibiotic treatment?	1, I had immediate improvement in my symptoms (<1 week)<br>2, I had gradual improvement in my symptoms (1-4 weeks)<br>3, I had improvement with long-term antibiotic use (more than 4 weeks)<br>4, I had no improvement in my symptoms<br>5, My symptoms got worse<br>6, My symptoms initially got worse, but improved over time<br>55, Can't remember<br>66, Not applicable
28. *[If participant had improvement of symptoms after taking antibiotics]* Did you experience a recurrence in the symptoms?	1, No<br>2, Yes, within 1 month of finishing the antibiotics<br>3, Yes, within 6 months of finishing the antibiotics<br>4, Yes, 6-12 months after finishing the antibiotics

	5, Yes, over a year after finishing the antibiotics<br>55, Can't remember
29. Were you diagnosed with any of the following co-infections or associated infections? *Check all that apply*	1, Babesiosis (Babesia microti, B. duncani)<br>2, Anaplasmosis (Anaplasma phagocytophilum)<br>3, Ehrlichiosis (Ehrlichia chaffeensis, other Ehrlichia species)<br>4, Bartonellosis (Bartonella henselae, B. quintana)<br>5, Mycoplasma (M. pneumoniae, other Mycoplasma species)<br>6, Chlamydia pneumoniae<br>7, Rickettsial disease (e.g. Rocky Mountain Spotted Fever/Rickettsia rickettsii, other Rickettsia species)<br>8, Other co-infection or associated infection (please specify)<br>44, None of the above<br>55, I don't know or can't remember
30. At what stage of infection was your Lyme Disease diagnosed?	Stage 1: Early Localized (roughly ≤1 month after infection; single EM skin lesion; infection has not yet spread beyond the skin)<br>Stage 2: Early Disseminated (roughly <3 months after infection; earlier spread of bacteria beyond a single EM skin lesion, e.g. multiple EM rashes, facial paralysis, acute meningitis, acute heart rhythm problems)<br>Stage 3: Late Disseminated (roughly 3+ months after infection; later stage of bacterial spread beyond the skin; any of multiple late manifestations, including arthritis, late neurologic manifestations, etc.)<br>55, I don't know or can't remember
31. Have you ever had any cord tissue, placental tissue, breast milk, cord blood, or fetal tissue tested for *Borrelia burgdorferi* (main bacterium that causes Lyme Disease in North America)?	1, Yes<br>0, No<br>55, I don't know or can't remember

SECTION 2: SUSPECTED LYME DISEASE AND TICK BITE HISTORY

[PARTICIPANTS WILL SKIP SECTION IF THEY HAVE EVER BEEN DIAGNOSED WITH LYME]

Questions	Responses
To be completed by participants who have NOT been formally diagnosed with Lyme Disease, whether suspected or not.	
1. Do you suspect that you currently have or have ever had Lyme?	1, Yes<br>0, No<br>2, I don't know
2. Have you ever had a tick removed from **your** body at any time?	1, Yes<br>0, No<br>2, I don't know<br>*[If no, skip to SECTION 3]*
3. *[If yes to having tick removed]* Approximately when was the tick removed?	MM/YYYY
4. *[If yes to tick being removed]* Was the removed tick submitted for testing for Lyme Disease?	1, Yes<br>0, No<br>55, I don't know or can't remember
5. *[If yes to tick being submitted]* Was the species of tick identified?	1, Yes<br>0, No<br>2, Unknown (did not receive results)<br>55, Can't remember
6. *[If yes to tick being identified]* What was the species of tick that was identified?	1, Lone star tick (*Amblyomma americanum)*<br>2, Black legged tick (*Ixodes scapularis)*<br>3, Western black-legged tick (*Ixodes pacificus)*<br>4, Ixodes angustus<br>5, Castor bean tick (*Ixodes ricinus)*<br>6, Woodchuck / groundhog tick *(Ixodes cookei)*<br>7, Brown dog tick (*Rhipicephalus sanguineus*)<br>8, American dog tick (*Dermacentor variablis*)<br>9, Gulf coast tick (*Amblyomma maculatum*)<br>10, Other (please specify)<br>11, Unknown (did not receive results)<br>55, Can't remember
7. *[If yes to tick being submitted]* Did the tick test positive for Borrelia burgdorferi (main bacterium that causes Lyme Disease in North America)?	1, Yes<br>0, No<br>2, Unknown (did not receive results)<br>3, Lab refused to test<br>55, Can't remember

8. *[If yes to tick being submitted]* Did the tick test positive for any of the other following bacteria? *Check all that apply*	1, *Anaplasma* spp.<br>2, *Babesia* spp.<br>3, *Bartonella* spp.<br>*4, Borrelia afzelii*<br>*5, Borrelia garinii*<br>6, *Borrelia mayonii*<br>*7, Borrelia bissetti*<br>8, Relapsing fever *Borrelia* (incl. *B. hermsii, B. perkerii, B. turicatae, B. miyamotoi, B. Lonestari*)<br>*9, Ehrlichia* spp.<br>10, *Rickettsia* spp.<br>11, Other (please specify)<br>12, Unknown (did not receive results)<br>13, Tick was not tested for these bacteria<br>55, Can't remember
9. *[If yes to having tick removed]* Did you develop an erythema migrans rash at the site of tick removal?	1, Yes<br>0, No<br>55, I don't know or can't remember
10. *[If yes to EM rash]* Approximately how many days after tick removal did the rash develop?	##
11. Have you experienced any of the following symptoms of Lyme Disease? Check all that apply	1, Fever<br>2, Recurring headaches<br>3, Recurring neck pain<br>4, Swelling of joints<br>5, Joint pain<br>6, Muscle pain<br>7, Fatigue<br>8, Facial drooping<br>9, Numbness and tingling<br>10, Muscle spasms or twitches<br>11, Dysfunction of the brain (including word retrieval and memory issues)<br>12, Lack of joint/muscle stability or function<br>13, Other Lyme Disease symptoms not listed (please specify)<br>44, None of the above<br>55, Can't remember
12. Approximately when did your Lyme Disease symptoms start?	MM/YYYY
13. Have you ever been tested for Lyme Disease?	1, Yes<br>0, No<br>55, I don't know or can't remember

14. *[If yes to every being tested]* How were you tested for Lyme Disease? *Check all that apply*	1, Blood test<br>2, Urine test<br>3, Joint tap<br>4, Cerebrospinal fluid (“spinal tap”)<br>5, Other (please specify)<br>55, I don’t know or can’t remember
15. *[If participant selected blood test]* Which blood test was performed? *Check all that apply*	1, ELISA / EIA (IgM/IgG)<br>2, Western Blot or immunoblot (IgM/IgG)<br>3, Two-tiered serology (ELISA / EIA + reflex Western blot)<br>4, PCR-DNA<br>5, T-cell response (EliSpot)<br>6, Other (Please specify)<br>55, I don’t know or can’t remember
16. *[For each blood test that was performed]* What was the result of this test?	1, Positive<br>0, Negative<br>2, Inconclusive (equivocal/borderline)<br>55, I don’t know or can’t remember
17. How do you suspect you were infected with Lyme Disease?	1, Tick bite<br>2, Congenital transmission (passed from mother to child)<br>3, Sexual transmission<br>4, Blood transfusion<br>5, Other (please specify)<br>55, I don’t know

SECTION 3: GSQ-30 SYMPTOMS OF LYME DISEASE

**GSQ-30 Symptoms**	**Responses**				
During the past 2 weeks, how much have you been **bothered** by any of the following?	0,<br>Not at all	1,<br>A little bit	2,<br>Somewhat	3,<br>Quite a bit	4,<br>Very much
1) Shortness of breath					
2) Feeling feverish					
3) Sweats and/or chills					
4) Nausea and/or vomiting					
5) Back pain					
6) Headaches					
7) Stiff or painful neck					
8) Muscle aches or pains					
9) Joint pain or swelling					
10) Muscle weakness					
11) Feeling fatigued or having low energy					
12) Feeling worse after normal physical exertion					
13) Trouble falling or staying asleep					
14) Needing more sleep than usual					
15) Not feeling rested on awakening					
16) Numbness or tingling					
17) Shooting, stabbing, or burning pains					
18) Skin or muscle twitching					
19) Discomfort with normal light or sound					
20) Balance problems or sense of room-spinning					
21) Change in visual clarity or trouble focusing					
22) Bladder discomfort or change in urination					
23) Light-headed or uncomfortable on standing					
24) Hot or cold sensations in extremities					
25) Irregular or rapid heart beats					
26) Feeling irritable, sad, or decreased pleasure					
27) Feeling panicky, anxious, or worried					
28) Trouble finding words or retrieving names					
29) Trouble with memory					
30) Slower speed of thinking					

Questions	Responses
Over the last 2 weeks, have any of the above impaired your work, social, or family functioning?	1, Yes<br>0, No

SECTION 4: PREGNANCY INFORMATION

Questions	Responses
1. Are you currently pregnant?	1, Yes<br>0, No
2. *[if YES to currently pregnant]* Other than your current pregnancy, how many times have you been pregnant?	##
3. *[if NO to currently pregnant]* How many times have you been pregnant?	##
**Current Pregnancy Module** *[if YES to currently pregnant]*	
1. When is the due date for this pregnancy?	DD/MM/YYY
2. During this pregnancy have you experienced any of the following? Check all that apply:	1, Vaginal spotting or bleeding<br>2, Extreme fatigue unresolved by rest<br>3, Trouble finding words or retrieving names, trouble with memory, slower speed of thinking<br>4, Exacerbation of joint pain or swelling<br>5, Bell's Palsy (sudden weakness or paralysis in facial muscles)<br>6, Hyperemesis gravidarum (severe nausea and vomiting that results in dehydration, weight loss, or ketosis)<br>7, Irritable uterus (frequent mild contractions that do not produce a change in the cervix occurring throughout pregnancy)<br>8, Prodromal labour (frequent mild contractions that do not produce a change in the cervix just prior to active labour)<br>9, Fever without a known cause<br>10, Pruritic urticarial papules and plaques of pregnancy (PUPPPs or "rash of pregnancy"<br>11, Preterm labour<br>12, Pre-eclampsia or pregnancy-induced hypertension<br>13, Gestational diabetes<br>14, HELLP syndrome (hemolysis, elevated liver enzymes, low platelets)<br>44, None of the above
**Other Pregnancy Module** *[if number of pregnancies/pregnancies other than current is at least 1. Additional modules for each additional pregnancy]*	
1. What was the outcome of this pregnancy?	1, Live birth<br>2, Miscarriage (pregnancy loss <20 weeks)<br>3, Stillbirth (pregnancy loss ≥20 weeks, delivery of a baby that has died in the womb)<br>4, Abortion
2. During this pregnancy did you experience any of the following? Check all that apply:	1, Vaginal spotting or bleeding<br>2, Extreme fatigue unresolved by rest

	3, Trouble finding words or retrieving names, trouble with memory, slower speed of thinking<br>4, Exacerbation of joint pain or swelling<br>5, Bell's Palsy (sudden weakness or paralysis in facial muscles)<br>6, Hyperemesis gravidarum (severe nausea and vomiting that results in dehydration, weight loss, or ketosis)<br>7, Irritable uterus (frequent mild contractions that do not produce a change in the cervix occurring throughout pregnancy)<br>8, Prodromal labour (frequent mild contractions that do not produce a change in the cervix just prior to active labour)<br>9, Fever without a known cause<br>10, Pruritic urticarial papules and plaques of pregnancy (PUPPPs, pregnancy hives "rash of pregnancy")<br>11, Preterm labour<br>12, Pre-eclampsia or pregnancy-induced hypertension (high blood pressure)<br>13, Gestational diabetes<br>14, HELLP syndrome (hemolysis, elevated liver enzymes, low platelets)<br>44, None of the above
3. *[For participants who have been diagnosed with LD AND answered "Yes" to question 31 in section 1]* For this pregnancy, did you have any of the following for tested for *Borrelia burgdorferi*? *Check all that apply*	1, Cord tissue<br>2, Placental tissue<br>3, Fetal tissue<br>4, Breast milk<br>5, Cord blood<br>6, Other (please specify)<br>44, None of the above
4. *[For each of the selected choices]* What was the result of this test?	1, Positive<br>2, Negative<br>3, Inconclusive (equivocal/borderline)<br>55, I don't know or can't remember
5. *[If outcome was miscarriage]* When did you miscarry?	MM/YYYY
6. *[If outcome was miscarriage]* How far along were you when you miscarried? (in weeks) *[dropdown options from 1-20]*	##
7. *[If outcome was live birth or stillbirth]* What was the gestational age at delivery (in weeks)?	##
8. *[If outcome was live birth or stillbirth]* When did you give birth?	DD/MM/YYYY
9. *[If outcome was live birth]* Child's weight at birth (in lbs and oz)	## lbs, ##oz
10. *[If outcome was live birth]* Child's biological sex at birth	1, Female<br>2, Male

11. Following this pregnancy, did you experience postpartum depression?	1, Yes<br>0, No
12. *[If pregnancy was a live birth]* Is this child still alive?	1, Yes<br>0, No
13. *[If child is not still alive]* How old was your child when they died?	*Open text*
14. *[If child is not still alive]* What was the cause of their death?	*Open text*

SECTION 5: CHILD HEALTH INFORMATION

Questions	Responses
**Child Health Module (for each pregnancy with a live birth)**	
1. Has this child ever been diagnosed with Lyme Disease?	1, Yes<br>0, No<br>55, I don't know or can't remember
2. *[If yes to diagnosis]* Was this child diagnosed with *congenital* Lyme disease?	1, Yes<br>0, No<br>55, I don't know or can't remember
3. *[If yes to diagnosis]* How was he or she diagnosed with Lyme Disease? *Check all that apply*	1, Clinical presentation of erythema migrans (EM) rash<br>2, Clinical diagnosis based on other (non-EM) symptoms/signs<br>3, Blood test<br>4, Urine test<br>5, Joint tap<br>6, Cerebrospinal fluid ("spinal tap")<br>7, Response to antibiotic therapy<br>8, Other (please specify)<br>55, I don't know or can't remember
4. *[If yes to diagnosis]* Approximately when was this child diagnosed with Lyme Disease?	MM/YYYY
5. *[If yes to diagnosis]* What symptoms did this child have that were attributed to Lyme disease by a doctor or other health care provider? *Check all that apply*	1, Erythema migrans (specify single or multiple)<br>2, Flu-like syndrome (e.g. fever, malaise, muscle/joint aches, headache)<br>3, Cardiac (e.g. heart block, arrhythmia)<br>4, Neurologic (e.g. facial or Bell's palsy, meningitis, meningoencephalitis, peripheral neuropathy)<br>5, Joint (e.g. arthritis)<br>6, Eye (e.g. optic neuritis, uveitis)<br>7, Borrelial lymphocytoma<br>8, Other (specify)<br>44, None of the above<br>55, I don't know or can't remember
6. *'[If yes to diagnosis]* Was your child diagnosed by a doctor or healthcare provider with any of the following coinfections or associated infections? Check all that apply	1, Babesiosis (Babesia microti, B. duncani)<br>2, Anaplasmosis (Anaplasma phagocytophilum)<br>3, Ehrlichiosis (Ehrlichia chaffeensis, other Ehrlichia species)<br>4, Bartonellosis (Bartonella henselae, B. quintana)<br>5, Mycoplasma (M. pneumoniae, other Mycoplasma species)<br>6, Chlamydia pneumoniae

	7, Rickettsial disease (e.g. Rocky Mountain Spotted Fever/Rickettsia rickettsii, other Rickettsia species)<br>8, Other co-infection or associated infection (specify)<br>44, None of the above<br>55, I don't know or can't remember
7. *[If no to diagnosis]* Was he or she ever tested for Lyme Disease?	1, Yes<br>0, No<br>55, I don't know or can't remember
8. *[If no to diagnosis]* Do you suspect that your child has, or has ever had Lyme Disease?	1, Yes<br>0, No<br>2, I don't know
9. Has this child ever had a tick removed from his or her body?	1, Yes<br>0, No<br>55, I don't know or can't remember
10. *[If yes to tick being removed]* Approximately when was the tick removed?	MM/YYYY
11. *[If yes to having tick removed]* Did he or she develop an erythema migrans rash at the site of tick removal?	1, Yes<br>0, No<br>55, I don't know or can't remember
12. Was this child ever breastfed or fed breast milk?	1, Yes<br>0, No
13. *[If yes to breastfed]* How old was this child when he or she COMPLETELY stopped breastfeeding or being fed breast milk?	1, Less than 1 week<br>2, 1 to less than 6 weeks<br>3, 6 weeks to less than 3 months<br>4, 3 months to less than 6 months<br>5, 6 months to less than 1 year<br>6, 1 to less than 2 years<br>7, 2 or more years<br>8, Child is still breastfeeding
14. Was this child ever fed formula?	1, Yes<br>0, No
15. *[If yes to formula]* How old was this child when he or she was FIRST fed formula?	1, Less than 1 week<br>2, 1 to less than 6 weeks<br>3, 6 weeks to less than 3 months<br>4, 3 months to less than 6 months<br>5, 6 months to less than 1 year<br>6, 1 to less than 2 years<br>7, 2 or more years
16. Was your child diagnosed with any of the following within the first two weeks of life? *Check all that apply*	1, Hyperbilirubinemia (jaundice)<br>2, Adenopathy (enlarged lymph nodes)

	3, Rash (please specify diagnosis)<br>4, Fever of unknown origin<br>5, Intrauterine growth restriction<br>6, Hypotonia<br>7, Respiratory distress<br>44, None of the above
17. Has your child ever been diagnosed with a congenital anomaly (“birth defect”)?	1, Yes<br>0, No
18. *[If yes to congenital anomaly]* Please specify which congenital anomaly. *Check all that apply*	1, Syndactyly (fingers or toes that are webbed or fused together)<br>2, Congenital heart defect (please specify)<br>3, Urological or Urogenital defect<br>4, Cleft lip and/or cleft palate<br>5, Neural tube defect<br>6, Other (please specify)
19. Has this child ever experienced any of the following musculoskeletal symptoms? *Check all that apply*	1, Joint pain or tenderness<br>2, Joint swelling<br>3, Bone Pain<br>4, Muscle Pain<br>5, Muscle twitching<br>44, None of the above
20. Has this child ever experienced any of the following gastrointestinal or urinary symptoms? *Check all that apply*	1, Swallowing difficulties<br>2, Nausea without known source<br>3, Recurrent abdominal pain without known source<br>4, Chronic constipation<br>5, Frequent diarrhea<br>6, Unexplained weight loss<br>7, Unexplained weight gain<br>8, Food intolerance (please specify)<br>9, Prolonged bed wetting<br>10, Frequent urination<br>11, Difficulty voiding urine<br>44, None of the above
21. Has this child ever experienced any of the following? *Check all that apply*	1, Night sweats<br>2, Excessive sweating<br>3, Sleep issues (please specify)<br>4, General fatigue<br>5, Difficulty concentrating<br>6, “Brain fog”<br>7, Limb or localized weakness<br>8, Dizziness

	9, Tingling/numbness<br>10, Palpitations<br>11, Sensory issues (i.e. sensitivity to noise, light, or touch)<br>12, Vision issues (please specify)<br>13, Colic<br>14, Failure to thrive<br>15, Hair loss or bald spots<br>16, Severe diaper rashes<br>17, Rashes or skin lesions (please specify)<br>18, Fever of unknown origin<br>44, None of the above
22. Has this child ever experienced any RECURRENT or PROLONGED infections of the following type? *Check all that apply*	1, Upper respiratory infections ("common cold"<br>2, Ear infections<br>3, Sinus infections<br>4, Strep throat<br>5, Other throat infections<br>6, Pneumonia<br>7, Other recurrent or prolonged infections (please specify)<br>44, None of the above
23. Has a doctor or other health care provider EVER told you that this child has any of the following? Check all that apply:	
a. ALLERGY/IMMUNOLOGY & HEMATOLOGIC (Blood disorders and conditions of the immune system)	1, Allergies (including food, drug, insect, or other)<br>2, Mast cell activation disease<br>3, ITP (immune thrombocytopenia or idiopathic thrombocytopenia purpura)<br>4, Elevated eosinophil count<br>5, Neutropenia (low neutrophil count)<br>6, Blood clotting or coagulation disorder (please specify)<br>7, Other blood abnormality (please specify)<br>8, Immunodeficiency (please specify)<br>9, Other autoimmune condition (please specify)<br>44, None of the above
b. ORTHOPEDIC/RHEUMATOLOGIC (Conditions of the bones, joints, and muscles)	1, Arthritis (please specify type)<br>2, Baker's cyst (behind knee)<br>3, Joint subluxation or dislocation<br>4, Joint hypermobility or Ehlers-Danlos syndrome<br>5, Plantar fasciitis<br>6, Knee issues (please specify)<br>7, Other orthopedic or rheumatologic condition (please specify)<br>44, None of the above

c. CARDIOVASCULAR/RESPIRATORY<br>(Conditions of the heart, lungs, and circulatory system)	1, Asthma<br>2, Postural orthostatic tachycardia syndrome (POTS)<br>3, Heart condition (other than congenital heart disorder)<br>4, Other cardiovascular or respiratory condition (please specify)<br>44, None of the above
d. FATIGUE, PAIN, OTHER MISCELLANEOUS SYNDROMES OR FUNCTIONAL DISORDERS	1, Chronic fatigue syndrome/Myalgic encephalomyelitis<br>2, Complex regional pain syndrome (also known as reflex sympathetic dystrophy)<br>3, Conversion disorder (blindness, paralysis, or other nervous system symptoms that cannot be explained by medical evaluation)<br>4, Functional abdominal pain<br>5, Functional neurologic disorder<br>6, Pain amplification syndrome<br>7, Psychosomatic disorder<br>8, Psychogenic non-epileptogenic seizures (PNES)<br>9, Somatoform disorder<br>10, Temporomandibular joint syndrome<br>11, Other fatigue, pain, or functional disorder or syndrome (please specify)<br>44, None of the above
e. NEUROLOGIC<br>(Conditions of the brain and nervous system)	1, Brain injury, concussion, or head injury<br>2, Benign intracranial hypertension<br>3, Chiari malformation<br>4, Frequent or severe headaches, including migraines<br>5, Epilepsy or seizure disorder (please specify)<br>6, Tourette syndrome or motor tics<br>7, Other neurologic disorder (please specify)<br>44, None of the above
f. GASTROINTESTINAL<br>(Conditions of the digestive system)	1, Celiac disease<br>2, Cyclical Vomiting Syndrome<br>3, Encopresis<br>4, Gastroesophageal reflux<br>5, Gastritis or eosinophilic esophagitis<br>6, Inflammatory bowel disease (e.g. Crohn's disease, ulcerative colitis)<br>7, Irritable bowel syndrome<br>8, Other gastrointestinal condition (please specify)<br>44, None of the above
g. DERMATOLOGIC<br>(Skin-related conditions)	1, Eczema<br>2, Psoriasis<br>3, Granuloma annulare<br>4, Pityriasis rosea<br>5, Other dermatologic condition (please specify)

	44, None of the above
h. ENDOCRINE<br>(Hormone-related conditions)	1, Adrenal insufficiency<br>2, Diabetes insipidus<br>3, Hyperthyroidism<br>4, Hypothyroidism<br>5, Polycystic ovarian syndrome<br>6, Other hormonal/endocrine condition (please specify)<br>44, None of the above
i. GENITOURINARY/RENAL<br>(Conditions of the kidney, urinary, or reproductive systems)	1, Interstitial cystitis<br>2, Vesicoureteral reflux<br>3, Recurrent urinary tract infections (UTIs)<br>4, Gynecologic problems (please specify)<br>5, Kidney problems (please specify)<br>6, Testes problems (please specify)<br>7, Other genitourinary condition (please specify)<br>44, None of the above
j. OCULAR<br>(Eye-related conditions)	1, Optic neuritis<br>2, Uveitis<br>3, Other eye condition (please specify)<br>44, None of the above
k. MENTAL HEALTH/DEVELOPMENTAL	1, Behavioural or conduct problems<br>2, Developmental delay<br>3, Intellectual disability<br>4, Speech or other language disorder<br>5, Learning disability<br>6, Autism or Autism Spectrum Disorder?<br>7, Attention Deficit Disorder (ADD) or Attention Deficit/Hyperactivity Disorder (ADHD)<br>8, Anxiety problems<br>9, Depression<br>10, Anorexia nervosa<br>11, Bulimia<br>12, PANDAS/PANS<br>13, Obsessive-compulsive disorder (non-PANDAS/PANS)<br>14, Any other mental health condition (please specify)<br>44, None of the above
l. OTHER	1, Gender dysphoria<br>2, Dental issues (other than occasional cavity) (please specify)<br>3, Parasite infection (please specify)<br>4, Other genetic or inherited condition (please specify)

	5, Other metabolic condition (please specify)<br>44, None of the above
24. Has this child ever experienced an adverse reaction following a vaccination?	1, Yes<br>0, No

SECTION 6: DEMOGRAPHICS

Questions	Responses
1. What is your month and year of birth?	MM/YYYY
2. Do you identify as racialized?	1, Yes<br>0, No<br>99, Prefer not to answer
3. What is your yearly household income?	1, Less than $20,000<br>2, $20,000-$34,999<br>3, $35,000-$49,000<br>4, $50,000-$74,999<br>5, $75,000-$99,000<br>6, Over $100,000<br>99, Prefer not to answer
4. How many people does this income support?	[Dropdown menu of number options 1-10+ plus option “Prefer not to answer”]
5. What is the highest level of school that you have completed?	1, Did not complete high school<br>2, High school diploma or equivalent (GED)<br>3, Some college, but did not complete diploma/certificate<br>4, College diploma/certificate<br>5, Some university, but did not complete degree<br>6, Bachelor’s degree<br>7, Graduate degree<br>99, Prefer not to answer
6. Do you have any of the following? Please check ALL that apply	1, Chronic Illness<br>2, Developmental Disability<br>3, Drug or Alcohol Dependence<br>4, Learning Disability<br>5, Mental Illness<br>6, Physical Disability<br>7, Sensory Disability (i.e. hearing or vision loss)<br>8, Other Disability (Please specify): ______________________<br>9, None<br>98, Do not know<br>99, Prefer not to answer
7. What country do you currently live in?	1, Canada<br>2, United States<br>3, Other (please specify)
8. *[If participant lives in Canada]* What province or territory do you currently live in?	1, Alberta<br>2, British Columbia

	3, Manitoba<br>4, New Brunswick<br>5, Newfoundland and Labrador<br>6, Northwest Territories<br>7, Nova Scotia<br>8, Nunavut<br>9, Ontario<br>10, Prince Edward Island<br>11, Quebec<br>12, Saskatchewan<br>13, Yukon<br>99, Prefer not to answer
9. *[If participant live in U.S.]* What state do you currently live in?	1, Alabama<br>2, Alaska<br>3, Arizona<br>4, Arkansas<br>5, California<br>6, Colorado<br>7, Connecticut<br>8, Delaware<br>9, D.C.<br>10, Florida<br>11, Georgia<br>12, Hawaii<br>13, Idaho<br>14, Illinois<br>15, Indiana<br>16, Iowa<br>17, Kansas<br>18, Kentucky<br>19, Louisiana<br>20, Maine<br>21, Maryland<br>22, Massachusetts<br>23, Michigan<br>24, Minnesota<br>25, Mississippi<br>26, Missouri<br>27, Montana<br>28, Nebraska

	29, Nevada<br>30, New Hampshire<br>31, New Jersey<br>32, New Mexico<br>33, New York<br>34, North Carolina<br>35, North Dakota<br>36, Ohio<br>37, Oklahoma<br>38, Oregon<br>39, Pennsylvania<br>40, Rhode Island<br>41, South Carolina<br>42, South Dakota<br>43, Tennessee<br>44, Texas<br>45, Utah<br>46, Vermont<br>47, Virginia<br>48, Washington<br>49, West Virginia<br>50, Wisconsin<br>51, Wyoming<br>99, Prefer not to answer
10. Do you live in an area where Lyme Disease is endemic?	1, Yes<br>0, No<br>98, I don't know
11. How did you hear about this study? *Check all that apply*	1, Facebook<br>2, Instagram<br>3, Twitter<br>4, Website<br>5, Email newsletter<br>6, From a friend<br>7, From a healthcare provider<br>8, Other (please specify)

SECTION 7: PATIENT PRIORITIES

[MODULE ONLY FOR PARTICIPANTS WITH SUSPECTED OR DIAGNOSED LYME]

Questions	Responses
1. How important to you is research on the each of the following?	1, Not important<br>2, Slightly important<br>3, Moderately important<br>4, Important<br>5, Very important
a) Lyme Disease in pregnancy	
b) Transmission of Lyme Disease in pregnancy	
c) Diagnosis and treatment of Lyme Disease in newborns	
d) Diagnosis and treatment of Lyme Disease in children	
e) Safety of breastfeeding with Lyme Disease	
2. Would you be interested in participating in future research regarding Lyme Disease and pregnancy?	1, Yes<br>0, No
3. *[If yes, show link to survey]* If you are interested in participating in further research, please click the following link and enter your contact information. Your responses will NOT be linked to this survey and will NOT be shared externally.	[LINK]
4. Is there anything else you think we should know regarding this survey or the topic of Lyme Disease in pregnancy?	*Open text*

Further Research Survey

If participants are interested in participating in further research, they will be directed to a separate survey with the following questions:

Questions	Responses
1. If you were pregnant, would you be willing to participate in a study that involved the collection of any of the following samples? *Check all that apply*	1, Breast milk<br>2, Cord blood<br>3, Placenta<br>44, None of the above<br>66, Not applicable
2. Would you be interested in participating in a follow-up focus group regarding future research into Lyme disease and pregnancy?	1, Yes<br>0, No
3. What is your email?	*Open Text*
