# Supplementary material for: Lyme borreliosis in pregnancy and associations with parent and offspring health outcomes: An international cross-sectional survey
Source: Front Med (Lausanne). 2022 Nov 3;9:1022766. doi: 10.3389/fmed.2022.1022766 (PMC9669415; doi:10.3389/fmed.2022.1022766)
Supplement: Supplementary file 3 [file Table_3.PDF]

Supplementary Table 3. Outcomes in children between the ages of nine and 22 by participant/parent LD status.

	Probable Treated LD Pregnancies n = 13	Probable Untreated LD Pregnancies n = 44	Possible Untreated LD Pregnancies n = 190	No Evidence of LD Pregnancies n = 137	p-value[a]
**Child age at parent participation, years (mean (SD))**	12 (3)	15 (4)	14 (4)	15 (4)	0.06
**Diagnosed with Lyme Disease**	46 (6/13)	49 (21/43)	51 (97/189)	18 (24/136)	<0.01
**Congenital Lyme disease**	83 (5/6)	71 (15/21)	56 (54/97)	0 (0/24)	<0.01
**Child age at LD diagnosis, years (mean (SD))**	2 (2)	10 (4)	10 (4)	13 (4)	<0.01
**Suspected Lyme disease**	15 (2/13)	26 (11/43)	29 (55/188)	18 (24/135)	0.10
**Any tick bite**	23 (3/13)	14 (6/42)	23 (43/187)	22 (30/136)	0.67
**Any musculoskeletal symptoms**	67 (8/12)	78 (31/40)	71 (128/180)	37 (49/131)	<0.01
**Any gastrointestinal/urinary symptoms**	83 (10/12)	85 (35/41)	81 (143/177)	53 (66/124)	<0.01
**Any recurrent infections**	45 (5/11)	76 (31/41)	67 (118/176)	52 (65/125)	<0.01
**Any nonspecific symptoms**	92 (11/12)	92 (37/40)	88 (159/180)	62 (81/131)	<0.01
**Night sweats**	17 (2/12)	35 (14/40)	31 (55/180)	11 (14/131)	<0.01
**Excessive sweating**	8 (1/12)	22 (9/40)	19 (35/180)	6 (8/131)	<0.01
**Sleep issues**	33 (4/12)	65 (26/40)	55 (99/180)	27 (36/131)	<0.01
**General fatigue**	67 (8/12)	52 (21/40)	55 (99/180)	24 (32/131)	<0.01
**Difficulty concentrating**	42 (5/12)	57 (23/40)	61 (109/180)	28 (37/131)	<0.01
**"Brain fog"**	17 (2/12)	42 (17/40)	47 (85/180)	17 (22/131)	<0.01
**Limb weakness**	17 (2/12)	28 (11/40)	23 (41/180)	11 (15/131)	0.03
**Dizziness**	8 (1/12)	38 (15/40)	34 (62/180)	15 (20/131)	<0.01
**Tingling/numbness**	25 (3/12)	35 (14/40)	26 (47/180)	14 (18/131)	<0.01
**Palpitations**	8 (1/12)	22 (9/40)	23 (42/180)	14 (18/131)	0.13
**Sensory issues**	67 (8/12)	60 (24/40)	54 (97/180)	23 (30/131)	<0.01
**Vision issues**	25 (3/12)	25 (10/40)	23 (42/180)	12 (16/131)	0.05
**Colic**	17 (2/12)	22 (9/40)	25 (45/180)	12 (16/131)	0.04
**Failure to thrive**	0 (0/12)	10 (4/40)	8 (14/180)	2 (3/131)	0.09
**Hair loss/bald spots**	17 (2/12)	15 (6/40)	4 (8/180)	5 (6/131)	0.03
**Severe diaper rashes**	8 (1/12)	22 (9/40)	18 (32/180)	6 (8/131)	<0.01
**Rashes or skin lesions**	33 (4/12)	32 (13/40)	24 (44/180)	13 (17/131)	0.01

**Fevers of unknown origin**	8 (1/12)	22 (9/40)	21 (37/180)	5 (7/131)	<0.01
**Any allergy/immunology/ hematologic diagnosis**	33 (4/12)	64 (25/39)	57 (99/174)	45 (57/126)	0.05
**Any orthopedic/ rheumatologic diagnosis**	40 (4/10)	44 (16/36)	38 (64/170)	19 (22/116)	<0.01
**Any cardiovascular/ respiratory diagnosis**	36 (4/11)	51 (19/37)	37 (59/160)	24 (28/119)	<0.01
**Any functional/ psychosomatic/pain diagnosis**	44 (4/9)	29 (10/34)	33 (51/154)	9 (10/117)	<0.01
**Any neurological diagnosis**	40 (4/10)	51 (18/35)	51 (83/164)	23 (28/122)	<0.01
**Any gastrointestinal diagnosis**	30 (3/10)	47 (18/38)	46 (72/157)	17 (20/118)	<0.01
**Any dermatologic diagnosis**	45 (5/11)	67 (24/36)	52 (85/164)	24 (28/119)	<0.01
**Any endocrine diagnosis**	10 (1/10)	24 (8/33)	18 (28/154)	10 (12/120)	0.11
**Any genitourinary/renal diagnosis**	10 (1/10)	17 (6/35)	22 (32/148)	13 (15/119)	0.26
**Any ocular diagnosis**	0 (0/8)	22 (8/36)	18 (26/148)	8 (9/118)	0.03
**Any mental health/ developmental diagnosis**	64 (7/11)	90 (37/41)	78 (139/178)	57 (73/129)	<0.01
**Other diagnosis**	11 (1/9)	44 (15/34)	36 (56/156)	17 (18/107)	<0.01
**Any adverse vaccine reaction**	25 (3/12)	20 (8/40)	32 (58/180)	18 (24/132)	0.03

Only includes live births with a known date of birth, where the child was between nine and 22 years old when their parent participated in the survey, and excludes pregnancies with "unclear" timing in relation to diagnosis/treatment/symptoms/tick bite. Values are percentages (n/N) unless stated otherwise.

[a] P-values calculated by Kruskal-Wallis rank sum tests (continuous data) and Fisher's exact tests (discrete data)
