# Supplementary material for: Lyme borreliosis in pregnancy and associations with parent and offspring health outcomes: An international cross-sectional survey
Source: Front Med (Lausanne). 2022 Nov 3;9:1022766. doi: 10.3389/fmed.2022.1022766 (PMC9669415; doi:10.3389/fmed.2022.1022766)
Supplement: Supplementary file 4 [file Image_1.PDF]

A)

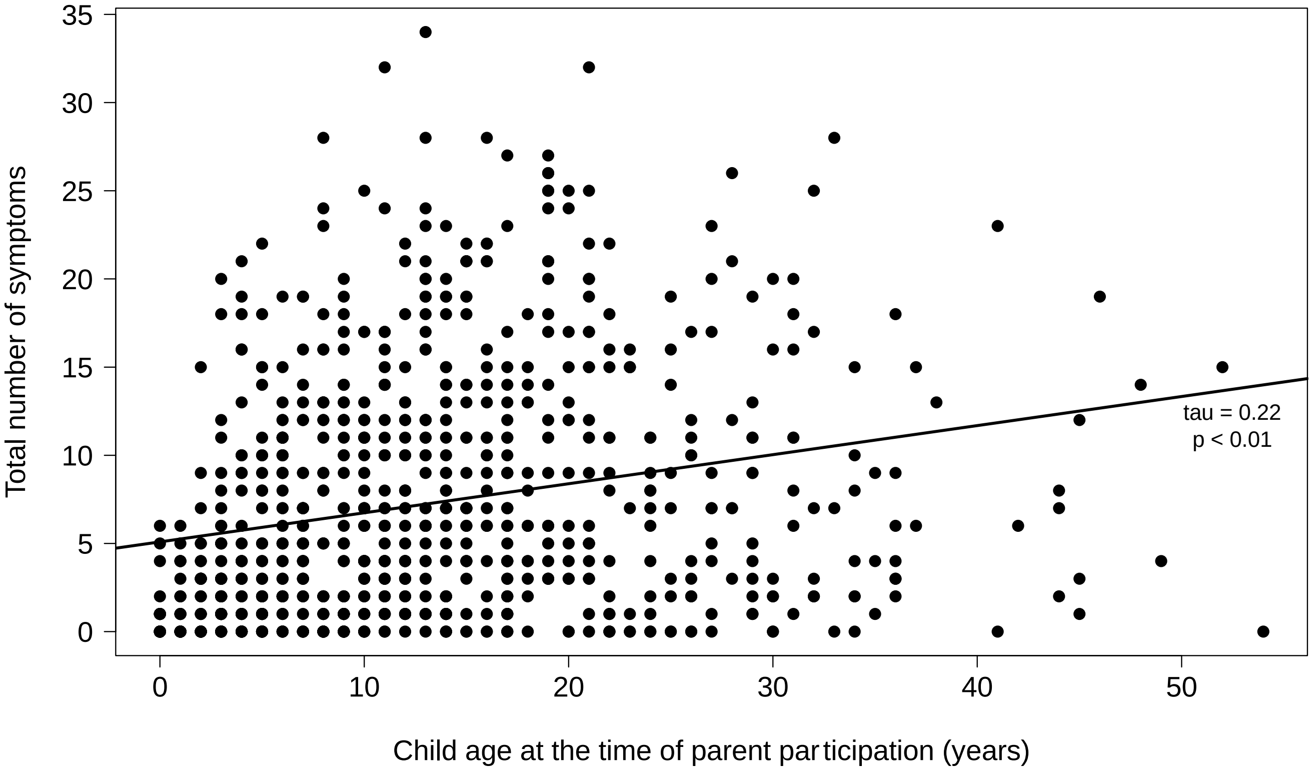

B)

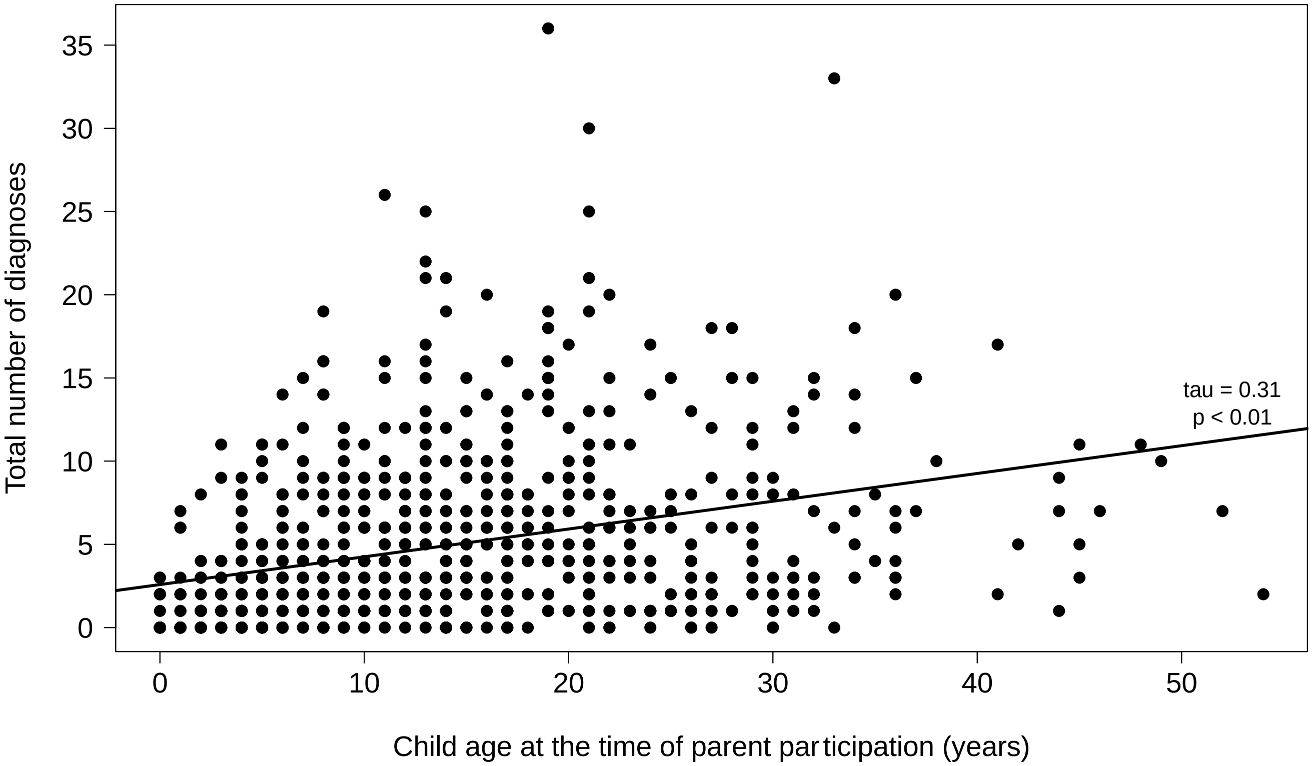

Supplementary Figure 1. Relationship between child age and overall health status. Children who were older at the time of their parents' participation in the survey study (x-axis) were noted as having more (**A**) health issues/symptoms (y-axis; tau=0.22, p<0.01) and (**B**) disease/disorder diagnoses by a medical professional (y-axis, tau=0.31, p<0.01).
